# The Inappropriate Symmetries of Multivariate Statistical Analysis in Geometric Morphometrics

**DOI:** 10.1007/s11692-016-9382-7

**Published:** 2016-04-18

**Authors:** Fred L. Bookstein

**Affiliations:** 1 University of Vienna, Vienna, Austria; 2 University of Washington, Seattle, WA USA

**Keywords:** Geometric morphometrics, Biological meaning, Multivariate statistical analysis, Covariances, Procrustes analysis, Deflation analysis, Morphometrics, Biomechanics

## Abstract

In today’s geometric morphometrics the commonest multivariate statistical procedures, such as principal component analysis or regressions of Procrustes shape coordinates on Centroid Size, embody a tacit roster of *symmetries*—axioms concerning the homogeneity of the multiple spatial domains or descriptor vectors involved—that do not correspond to actual biological fact. These techniques are hence inappropriate for any application regarding which we have a-priori biological knowledge to the contrary (e.g., genetic/morphogenetic processes common to multiple landmarks, the range of normal in anatomy atlases, the consequences of growth or function for form). But nearly every morphometric investigation is motivated by prior insights of this sort. We therefore need new tools that explicitly incorporate these elements of knowledge, should they be quantitative, to break the symmetries of the classic morphometric approaches. Some of these are already available in our literature but deserve to be known more widely: deflated (spatially adaptive) reference distributions of Procrustes coordinates, Sewall Wright’s century-old variant of factor analysis, the geometric algebra of importing explicit biomechanical formulas into Procrustes space. Other methods, not yet fully formulated, might involve parameterized models for strain in idealized forms under load, principled approaches to the separation of functional from Brownian aspects of shape variation over time, and, in general, a better understanding of how the formalism of landmarks interacts with the many other approaches to quantification of anatomy. To more powerfully organize inferences from the high-dimensional measurements that characterize so much of today’s organismal biology, tomorrow’s toolkit must rely neither on principal component analysis nor on the Procrustes distance formula, but instead on sound prior biological knowledge as expressed in formulas whose coefficients are not all the same. I describe the problems of the standard techniques, discuss several examples of the alternatives, and draw some conclusions.

## Introduction

Modern quantitative natural scientists are introduced so early in their training to the standard metaphorical structures of twentieth-century applied statistics—numerical variables and their linear combinations, their tabulation in matrices, and their correlations or covariances—that the scientific foundations of these practices are hardly ever subjected to close scrutiny. This essay is meant as a discipline-specific example of such an examination: how cogent the multivariate strategies might be that underlie a relatively new branch of biometrics, *geometric morphometrics* (GMM). I will conclude that its current choices of multivariate method suit its actual subject matter (the biologically meaningful analysis of Cartesian coordinates of homologous landmarks) so imperfectly as to invalidate many, perhaps most of the rhetorics by which its findings are typically reported.

The main problems that engage me are the inadequate information resources of the conventional matrix notation, the incoherence of the linear combinations that comprise the typical reporting language for patterns uncovered in the course of analyzing the matrices, and the difficulty of interpreting “rotations” of lists of variables, such as the conventional rotations to Procrustes shape coordinate space and then its principal components that supply the axes of most of GMM’s published scatterplots. These are explored in section “Four Ubiquitous Problems”. Section “Some Alternative Methods” reviews some partial resolutions of these paradoxes already available in the literature, and section “A-Priori Information to Break the Symmetries of GMM” lists some of the sources of symmetry-breaking information that are available to the theoretical biologist but that are not yet incorporated in any of our standard analytic maneuvers. Section “Two Evolutionary Examples” presents, in précis, two worked examples of the techniques envisioned here. A closing discussion, section “Discussion: Solutions Yet to Be Envisioned”, goes on to sketch an assortment of more radical possibilities. I hope some of these will eventually become the focus of newly energized methodological experiments aimed at altering the rhetoric of inference first in morphometrics and later in quantitative organismal biology more generally. Following this discussion are two Appendices. The first of these exemplifies the article’s critique in an application to one formula of current interest, the so-called *RV*-coefficient that ostensibly helps to report the relationship between two blocks of measurements on the same sample. The second is a detailed examination of the geometry by which Cartesian coordinates or their shape-coordinate cousins actually generate the covariances to be processed by principal components methods.

*Some philosophical preliminaries* As far as its actual formulas are concerned, this essay reduces to some rules of good practice that should govern the ways arithmetic is turned into understanding in the course of studies of organismal form. Some of its caveats are not specific to that organismal context, but instead overlap with what good practitioners of applied multivariate analysis already know, namely, that reliable prior scientific knowledge should logically dominate arithmetical rules, not vice versa. Yet I have been unable to locate any printed history of multivariate analysis in biology, let alone one that traces the privileged role of principal component analysis and other optimizing representations. (There is a brief review of the occasional earlier paragraph about that specific technique in Bookstein 2015c.)

The central desideratum on which my arguments focus, the furtherance of “biological meaning,” is one standard trope in the philosophy of biology. In its quantitative aspects I am averring mainly to a social phenomenon bracketed between two great students of twentieth-century practice, Ludwik Fleck and Edward O. Wilson. Writing in the 1930s, Fleck (1979) teased out an explication of scientific consensus, the *Denkkollektiv* (thought collective), that wholly anticipated Thomas Kuhn’s great insights about “paradigms” a long generation later. In an aphorism summarizing the original German thesis (Bookstein 2014:xxviii) this view runs, “A scientific fact is a socially imposed constraint on speculative thought.” Fleck’s example was the evolution of the understanding of syphilis: what brought about the coalescence of the modern view was the success of early twentieth century serology at showing the quantitative stability of the regressions relating symptoms to blood measures.

Wilson (1998) was conveying this same message when he resurrected William Whewell’s long-dormant notion of the *consilience* of the natural sciences, the anticipated convergence upon a common truth of lines of evidence from many directions. Wilson suggested that this be taken as the governing principle of *all* the sciences we might call “natural” (including, notoriously, human sociobiology): “Trust in consilience is the foundation of the natural sciences” (Wilson 1998:11; Bookstein 2014:29–30). The way numbers acquire meaning in the organismal biological sciences is by their potential role in producing consilience in this sense: *numerical* agreement (of actual value, not merely of an associated plus or minus sign) across a multitude of different ways a numerical signal might be probed (Bookstein 2014) while historical conditions, sample design, and experimental settings are all varied in turn. Thus consilience is a matter of systematically *altering* the instrumentation supporting a quantitative argument. As a homely example, it would be more persuasive to confirm the distance (in meters) a vehicle travels by the product of a directly measured speed, in m/s, times a directly measured elapsed time than simply by measuring displacement a second time with a different camera. As Collins (1985) puts it, persuasion in the sciences of complex organized systems arises mainly from the very careful control of replication across many levels.

According to this notion, the emergence of biological meaning as a community activity, it is the agreement of estimates of the same quantity from essentially different types of measuring instruments that makes some of the subdisciplines of biology, from genomics to evo-devo, into the quantitative natural sciences that, here in the twenty-first century, they are turning out to be. This essay is a collection of notes toward applications of geometric morphometrics that further that end. The collection emphasizes warnings against the *mis*applications, the mistaken arguments that purport to wield a computed number or pattern description as if it supported one selected interpretation (usually the author’s) far more strongly than it actually does.

I do not agree with Platt’s (1964) famous article (reprinted in Platt 1966) about the role of “strong inference” in biology. Platt says, referring to the context of discovery,

Measurements and equations are supposed to sharpen thinking, but, in my observation, they more often tend to make the thinking noncausal and fuzzy. They tend to become the object of scientific manipulation instead of auxiliary tests of crucial inferences. Many—perhaps most—of the great issues of science are qualitative, not quantitative, even in physics and chemistry. Equations and measurements are useful when and only when they are related to proof; but proof or disproof comes first and is in fact strongest when it is absolutely convincing without any quantitative measurement. Or to say it another way, you can catch phenomena in a logical box or in a mathematical box. The logical box is coarse but strong. The mathematical box is fine-grained but flimsy. The mathematical box is a beautiful way of wrapping up a problem, but it will not hold the phenomena unless they have been caught in a logical box to begin with.

I am not being so demanding here. (Perhaps organismal biology has not yet reached its Golden Age, about which Platt was reminiscing so nostalgically from the molecular-biological point of view 50 years ago.) It is quite possible that meaningful insights *can* emerge from the careful study of empirical organismal patterns relating multiple measurements under carefully controlled conditions of observation along with suitably elegant arithmetic. Biomechanics is generally consistent with classical kinematics, continuum mechanics, hydrodynamics, and aerodynamics; population genetics is consistent with classical probability theory in many ways; even the cognitive neurosciences *may* prove consistent with information theory and, reading backward, the classical thermodynamics of entropy and free energy (Friston 2010). Yes, many of our mathematical foundations can be borrowed from these more seasoned domains of quantification. What we borrow are often the quantities that those other fields reassure us are the ones most worth recording: biomass and bioenergetics, chemical gradients, stable molecular arrangements like membranes or the double helix. At the same time, other fields, such as comparative anatomy, seem just as far from a satisfactory quantitative foundation here in 2016 as they were 40 years ago when I was just beginning my work. It cannot hurt to point out these divergences.

Indeed there is a surprising dearth of literature about the foundations of measurement in organismal biology. The biophysicist Walter Elsasser, writing in the twilight of his career, refers to the biologist’s focus on “holistic memory,” meaning, memory without storage (Elsasser 1988:42–43), as the aspect in which biology most diverges from the other natural sciences. But any possibility of specific insight seems to be inaccessible, only the general adviso that one needs to measure only a few very carefully selected aspects of the incomprehensibly high-dimensional state space that any organism actually occupies (Elsasser 1975:203). To say measurement requires forethought is not a trivial point even if one finds it very often trivialized in the papers of the GMM tradition when they say, at the very beginning, “Here are my landmarks,” without any justification from the explanations to which those landmarks are supposed to contribute and usually without any evidence that the organism cares about those locations in any systems-maintenance sense.

This issue, so central to the general run of the other natural sciences, is oddly absent from our field’s standard treatises. Frequently cited classic references will often fail to place any logical or biological requirements on the relevance of the number line to whatever point is being made about a formula for a path analysis, or a parent-child covariance, or whatever. The role of statistical formulas is thereby misleadingly rendered as if somehow independent of the content of the variables whose numerical values are being thereby transformed or transcribed using implicitly reductionist arguments from chemical kinetics, energetics, kinematics, or scaling. In this way formulas like the correlation coefficient or the regression coefficient cease to be aspects of the science we are pursuing, but stand instead for lazy metaphors: rhetorical tropes the foundations of which go generally unexamined. This essay examines those foundations for a few of these most fundamental metaphors.

It is ironic to contrast this inattention with the far greater importance that issues at the foundation of analogous quantifications bear in the psychological sciences (e.g., Coombs 1964; Krantz et al. 1971–1990) or even in the economic sciences (Morgenstern 1950). The biologist often behaves as if any convenient quantitative score extractable from an organism is *ipso facto* the kind of number regarding which one can legitimately carry out the sort of elementary statistics we teach our beginning graduate students: the kind of number that can be averaged over convenient samples of specimens, squared and converted to variances or their components, multiplied so as to be converted to covariances or correlations, converted to a probability in the course of setting down a discriminant function, etc. But even to state such an assumption is to highlight how unreasonable it must be in most empirical contexts.

Our literature offers even less discussion of the meaning of characterS, plural: their assembly into “data matrices.” Here is more or less everything that Sewall Wright has to say about “the importance of choice of variables” in Chapter 6 (“Types of Biological Frequency Distributions”) of volume 1 (1968) of his masterpiece *Evolution and the Genetics of Populations*:

It is probably usually true that measures of volume or weight, whether of the organism as a whole or of some one organ, associated with appropriate indexes of form are more instructive than linear measurements. On the other hand, indexes must be based on measurements and their use involves certain statistical pitfalls.

Wright goes on with a full-page five-panel offering of “some unimodal distributions of indexes,” Figure 6.4, all of which are ratios of pairs of length or area measures. We are evidently a very long way from geometric morphometrics here. Later, in Chapter 4 (“Variability under Inbreeding and Crossbreeding”) of volume 3 of the same treatise, the measurements in the examples are all either extents (length, weight) or concomitants of fitness (litter size, percent liveborn). None of his examples seem to involve measurements of geometric shape, the core concern of contemporary morphometrics.

Lande (1979) likewise seems to be limiting his attention to the case of two measures of extent, as shown by the tail of his title, “$\ldots$ applied to brain:body size allometry.” It is clear that his methods apply only to such measures of extent because he refers to Huxley’s (1932) method of loglinear regression, counseling that “characters [should be] measured on scales such that the intraspecific phenotypic variances are roughly constants; $\ldots$ for metrical variables this can usually be accomplished by employing logarithmic scales.” Of course the shape coordinates produced by today’s best GMM analyses are not positive quantities—they must, for instance, average zero along each of four entirely different dimensions—and so cannot be log-transformed. The advice one gets from the population genetics literature, whether classic (Lande, Wright) or contemporary (Felsenstein’s publicly posted book draft of 2015, in which every covariance coefficient deals with some single measurement undergoing a comparison across relatives), evidently is not meant to apply to more general schemes for quantifying organismal form, such as those of this paper. At least, I can find no evidence that such schemes have ever been adequately theorized.

My context here in this essay is geometric morphometrics (GMM), not biometrics in general, and it is multivariate, dealing with characters in lists rather than one by one. We will see that some of the problems that ensue are with the “G” of GMM, while others deal with the “MM” component. So the covariances between (mid)parents and their offspring are not among the examples I have in mind—not if the values (like $1\over 2$) they are intended to match are integer fractions derived from formulas instead of other data. Likewise the paths along which these covariances, once normalized, turn into regressions are intended to be real morphogenetic paths capable of experimental confirmation or perturbation: causal relations that can be modified in an experimental setting by changing some controllable aspect of epigenesis or function. Covariances between measures of form and calendar dates rarely meet this criterion (but they can, as when we study experimental modifications of the life cycle itself, as with farmed salmon); likewise, at least in biology, studies in which the regressor has units of thousands or millions of years. Whenever a regression slope comes in units of $u_1/u_2,$ as is the case for an automobile’s speedometer, there ought to be a way of estimating the slope by a direct instrument measurement rather than by merely replicating the ratio of measured rise to measured run from which it originally derived.

There may be a particular problem with the language of genomics vis-a-vis this multivariate setting. (For the sentiment of this paragraph I am deeply indebted to multiple conversations with my Vienna colleague Philipp Mitteroecker.) For example, the word “additivity” and its complement, “dominance,” do not seem to extend at all well into the present context. Kenney-Hunt and Cheverud (2009) noted that, generally, speaking, morphospace is an uncomfortable setting in which to indulge the rhetoric of population genetics in that it is more or less guaranteed you will find overdominance no matter what processes actually produced the data on which you are relying. Their claim is one version of my Shape Nonmonotonicity Theorem (Bookstein 1980), which basically states that in any geometric morphospace of more than two landmarks, for any three forms A, B, and C you might name there will be an indefinite range of empirical variables for which A and B score the same whereas C’s score is different. That theorem, in turn, is a special case of the version in Bookstein (2002), an equally insidious challenge to the role of intuitive pattern claims in multivariate biometrics, that for just about any collection of $2k-3$ specimens or fewer on *k* or more landmarks, and for any separation of the list of $2k-3$ into two exclusive subsets A and B, one can construct a shape measure for which all the specimens of A have one score and all the specimens of B have a different score, without any within-“group” variance on either side. Only if the biology constrains that coordinate for you in advance—only if the symmetries about which this essay is complaining have been superseded by strong prior knowledge of mechanism—does it make any sense to apply univariate arithmetical terms like “additivity” to multivariate population-genetic data.

But, really, the problem is not specific to particular subdisciplines of the organismal sciences. The lack of a foundation for turning arithmetic into biological understanding of organismal form is at root the lack of a foundation for the way we generate quantitative descriptions *of* that form. Geometric morphometrics is based on just such a protocol for the generation of such descriptions. Let us see to what extent and in what contexts of study design the advice it proffers us on sound method can prove constructive rather than destructive.

## Four Ubiquitous Problems

Several ubiquitous problems of multivariate analysis in geometric morphometrics arise from the fact that its foundations in biology seem never to have been properly established. Three of these are the vacuity of matrix notation, the fugitive meaning of linear combinations of measurements or coordinates, and the nonbiological nature of multivariate rotations; and one special case of these rotations, our routinized Procrustes analysis, deserves a subsection of its own.

### Matrices

“Matrix notation,” as everybody knows, reifies a rectangular array of numbers by ordinating its contents in a double-subscript scheme. An array $(a_{ij}),$ where each single $a_{ij}$ is the number in the *i*-th row and *j*-th column, is considered to represent a single conceptual object, “the matrix *A*,” for purposes of many multivariate pattern analyses and the associated quantitative styles of biological inference. For example, the data matrices with which GMM is most concerned are matrices of *Procrustes shape coordinates,* which are carefully normalized Cartesian locations of digitized landmarks or semilandmarks (standardized samples from curves or surfaces). The rows of *A* are now individual specimens, while the columns are coordinates of the landmarks that were gathered. (See section “Procrustes Distance, Procrustes Coordinates”.)

**Fig. 1:**
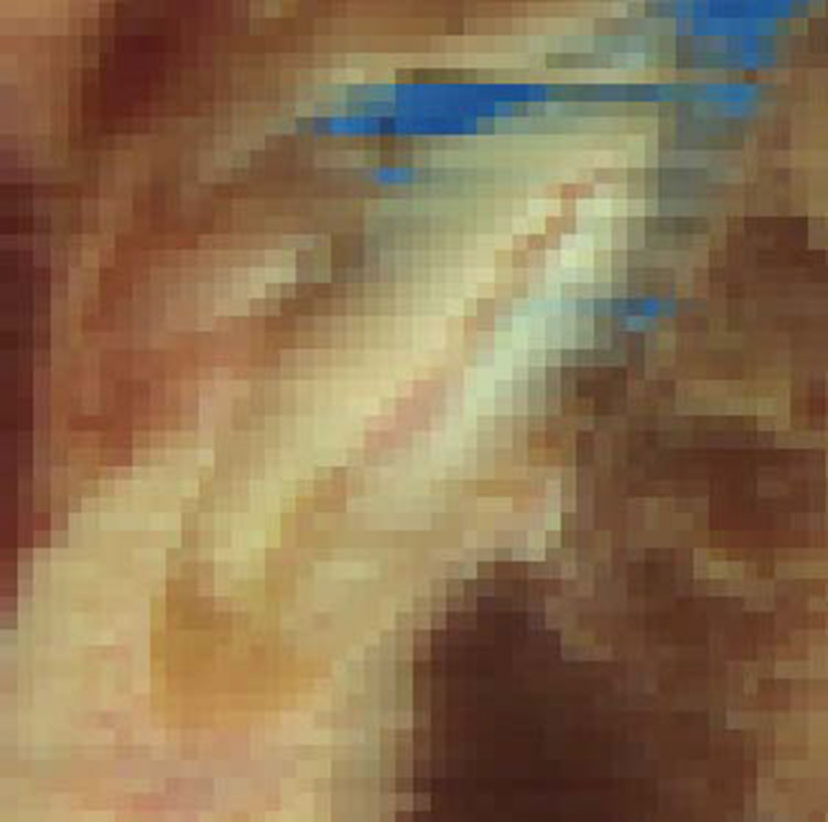
A digital image is an unusually tractable kind of matrix in that row number, column number, and subscript-to-subscript Euclidean distance all have physical interpretations. This example is a very small synthetic slice of the *full-color* image of the NLM Visible Female (“Eve”): a medial section of one of her central lower incisors, with its canal, in the jawbone. This is a real image, not a virtual one, and it is realistically noisy. *Colors* are those of the original tissues except that *blue* represents the latex used to fix movable structures (here, the teeth themselves) against the forces exerted by the microtome, the forces that are also responsible for the left-to-right smearing in some portions of the image. Original sections were horizontal at spacing $300\,\upmu$, photographed with pixel size also $300\,\upmu$ in order to yield cubical voxels. Image produced in W. D. K. Green’s Edgewarp software package. The original image is $5180 \times 960 \times 1664 \times 3,$ about 24 gigabytes; the three thousand or so pixels of this extract are thus a very small selection (Color figure online)

Consider those subscripts *i* and *j*, $i=1\ldots n$ for rows, say, and $j=1\ldots p$ for columns, a bit more carefully. We know a little in advance about these two lists. For instance, as printed they arrive in a natural order, the order of the “natural numbers” (the integers). Any index *i* for rows or columns lies in-between any index $i-k$ preceding it and any index $i+l$ following it. For the matrices representing images, this might be all we need to know. For example, one gets “regions” of those images by agglomerating entries $a_{ij}$ for which the corresponding subscript pairs *ij* are near neighbors in some suitable sense. (This is the case, for instance, for the pixels in Fig. 1.) Or it might be the case that the order of one of these subscript lists makes sense even if the other doesn’t: specimens that were observed at an ordered series of ages, for instance, on an unordered list of properties (weight, coat color, brand of chow, behavior). Or specimens might have a hierarchical structure: five from group A, six from group B, $\ldots$ Conventionally all this is encoded via a list of additional columns of the matrix, *dummy variables,* that at least can accompany the data set on its way to our favorite software package.

**Fig. 2:**
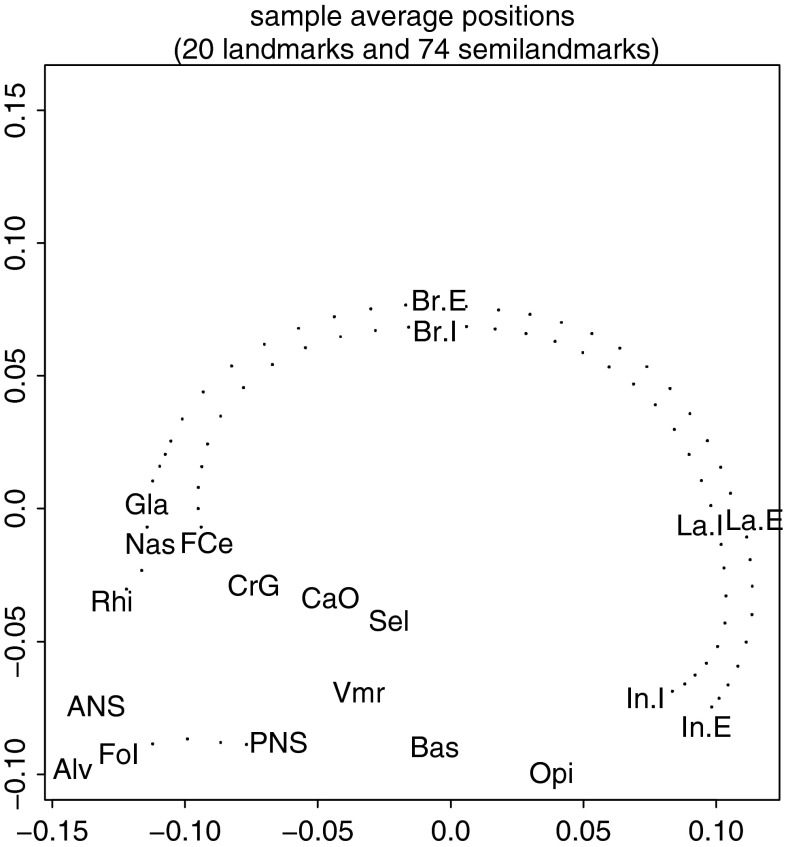
A typical template, this one corresponding to the left-facing two-dimensional hominid calva example in Bookstein (2015b). The 20 abbreviations correspond to 20 landmarks, the 74 dots to semilandmarks arbitrarily spaced on arcs connecting some of the landmarks in pairs

But it is much more common in organismal biology in general, and in morphometrics in particular, for there to be a far more intricate order among the variables than can be represented simply by reference to integers. GMM’s landmarks, for instance, have adjacencies just like the pixels of Fig. 1 did—but those adjacencies are not gridded the way subscripts are: they are functions of the column (coordinate) means, in pairs (2D data) or triples (3D data), not the subscripting scheme per se. For 3D data, the matrix notation can handle neither the conceptual orthogonality of the *x*, *y*, and *z* directions nor this structure of successive triples pertaining to the same point. As far as the matrix is concerned, the column representing the *x*-coordinate of landmark 1 is considered to potentially relate in the same way to the column representing the *y*-coordinate of landmark 1 as it does to the *x*-coordinate of landmark 2, or the *y*-coordinate of landmark 2, for that matter. It is a lot of work for subsequent algorithms to recover from an ignorance so profound about the kind of information that is visually so obvious in diagrams of a *digitizing template* such as the example in Fig. 2.

In schemes like this, landmark points bear two proper Cartesian coordinates, and often they indicate boundaries between tissues or other functionally or morphogenetically relevant information. But the semilandmarks (here, those unnamed dots) record arbitrarily spaced information from curving form in-between, and so while one of their coordinates (the one normal to the curve) incorporates quantifiable information about extent, the other encodes a different kind of information, about tangent direction. If this information is to be relevant to comparative explanations (as it often is, in the study of joint articulations, for example), it must be via a different formalism than the Procrustes analyses we will consider in section “Procrustes Distance, Procrustes Coordinates”. We will learn more about the handling of these issues of ontology and spacing in section “Deflated Procrustes Analysis”. For now, it is enough to remark that the necessary information is missing from the matrix record itself as it currently stands. An analogous opportunity connected with the origin of certain lists of specimens in a branching history (a phylogeny) supplies the impetus for the comparative approach that will concern us at section “The Comparative Method for Analysis of Contrasts Across a Phylogeny” and again in section “Modifying a Comparative Analysis of Mammalian Skulls”.

Even when variables are spaced along only one dimension—time, perhaps, or spectral frequency—we need additional information beyond the subscript *j*, the column number: we need to know the numerical value of the instrumental setting we were are using at the time this particular column of data was collected. “Each animal was measured at age 7 days, 14, 21, 30, 40, 60, 90, and 150,” we might be informed, or, “acoustic energy was assessed in each of the following eight frequency bands: $\ldots$ ” When variables are associated with the settings of dials on machines for signal-filtering or image capture, that information likewise must accompany the matrix accommodating the vectors of readings specimen by specimen; but in none of these cases is such notation available to the matrix calculator.^1^ And even for the GMM data resources somewhat less information-rich than the coordinate or spectral records—matrices of measured lengths—there is still considerable information missing, about the location of those distances upon the typical form and their subdivision into rigid, elastic, or articulated components. See the analysis of Wright-style factor analysis, section “Sewall Wright’s Style of Factor Analysis”.

Wherever in the course of subsequent sections we are able to claim *any* cogency for the methodological adjustments demonstrated or proposed, it can only be because an analytic tactic has been uncovered that modifies standard matrix calculus approaches in order to accommodate the information that would otherwise have gone missing. Often this is the information about logical connections among the rows or columns, in pairs or longer sublists, that is intentionally omitted from the *ij* subscripting scheme for the matrix content itself.

### Linear Combinations

Just as we are used to matrix notation, numbers in rows and columns, we are likewise used to the notation of linear combinations of variables, formulas like “$b_1X_1 + b_2 X_2 +~ \cdots ~+ b_pX_p.$” It should not be as rare a cognitive stance as it actually is to step back from this sort of formula for a moment and ask about the biological meaning of its elements: the “coefficients” $b_i,$ the “variables” $X_i$ being agglomerated, and especially the operator “+” (or “−” if you change the sign of the coefficient) that is taking the responsibility for the arithmetic here.

We can easily imagine nonsense examples of this notation: formulas like “5$\times$ humerus.length—3$\times$ aortic.valve.angle.” We must demand at the outset that at least the units of quantities being combined by a plus or minus are commensurate: one cannot add centimeters to radians. Let us edit the example, then, so that it now reads “5$\times$humerus.length—3$\times$aortic.valve.length.” But of course much more is required. If the arithmetic result is to be a predictor of some exogenous quantity, it needs to come in a unit of its own, say, grams (or perhaps a composite unit such as dynes, gm cm/s$^2$). Then the coefficients 5 and $-3$ must each be in units of grams per centimeter (or grams per second per second), and one of the two principled ways to generate vectors of coefficients of additive combinations like these is as multiple regression coefficients, or, as the geneticist Sewall Wright renamed them, *path coefficients.* (The other way, also identified with Sewall Wright, is his approach to general and special factors, section “Sewall Wright’s Style of Factor Analysis”. In that context, the coefficients of the linear combinations specify effects, not causes.)

Regression coefficients, in general, arise from multiple causal pathways in play at the same time. Their assumptions must be minutely examined whenever such a linear combination is written down. Each coefficient must apply to the expected effect of change in one predictor regardless of the values of any of the other predictors, and the effect of, e.g., raising humerus length by 2 units must be equal and opposite to the effect of lowering it by the same two units, *regardless of its current value.* Such assumptions are nearly impossible to verify in any real data set, and in their absence it is unjustified to believe in the reality of any process calibrated by the coefficient vector of *b*’s under scrutiny. And what, in general, do we make of the fact that some components of the summation are positive and some are negative? Does the process we are studying even *allow for* interventions that differ in sign? Physiological parameters, in particular, must be positive; kinetic energy, likewise; one cannot lower ambient water pressure or the jaw gape of a predator past zero. There will be more to say about linear combinations when we discuss consequences of the Perron–Frobenius theorem at section “Sewall Wright’s Style of Factor Analysis”.

Linear combinations are even more problematic when the variables being combined are Cartesian coordinates. In that setting, the formula must combine terms in all coordinates for all the landmarks. The arithmetic, then, looks like “$a_{1x}X_1+a_{1y}Y_1+a_{1z}Z_1+a_{2x}X_2+\ldots ~.$” In this setting the symbols $+$ and − stand for *directions in the coordinate space.* If $a_{1y}$ is positive, for instance, its positivity means that the picture of this component will involve a shift of $a_{1y}$ in the direction of increase of the *y*-coordinate of the first landmark along with analogous shifts in every other coordinate at the same time. (There may also be a thin-plate spline grid following these shifts along, the better to see their regional organization.) Evidently we are not talking about arithmetic, $+$ and −, but about *vectors,* shifts of the first landmark in the direction $(a_{1x},a_{1y},a_{1z})$ at the same time that every other landmark is being shifted according to *its* little three-vector. To interpret the original expression $a_{1x}X_1+a_{1y}Y_1+a_{1z}Z_1+a_{2x}X_2+\ldots$ as an actual number is to presume that it is biologically meaningful to “project” any observed composite shift of all the landmarks at once against this particular direction in their common vector space. But such a projection presumes the meaningfulness of the geometric aspects (shortest distances, or, equivalently, perpendicularity of the residual to the projection) that treat all directions as somehow equivalent in their potential biological meaning. Hence the concern for linear combinations of coordinates is inseparable in principle from a worry about the meaning of their directions, which is to say, the structure of rotations between directions or sets of directions in these spaces of linear combinations.

### Rotations, Especially Their Basis in Covariance Structures

Rotations can be thought of as a special case of the preceding, when a whole list of linear combinations of the same *X*’s is considered at once such that the coefficients of each linear combination have zero crossproduct with the coefficients of any other and individually sum in square to 1.0. This is the characterization of the *orthonormal transformations* that leave pairwise interspecimen Euclidean distances $\Sigma _j (X_{{i_1}j}-X_{{i_2}j})^2$ invariant. The statistically minded organismal biologist almost never pauses to contemplate the fact that corresponding to these criteria—sums of products of coefficients, “distances” between specimens—*there is no biology at all.* In the formula for distance, why should different variables $X_i$ enter with equal weights? In the formulas for the rotations, why should the organism care if linear combinations are orthogonal?

Of the two most commonly encountered settings in which rotation is invoked in GMM, one is the rotation to *principal components.* (The other, the rotation that constitutes the Procrustes fit itself, is dealt with in the next subsection.) In principal components analysis, which when applied to shape coordinates is usually called *relative warps analysis,* the linear combinations that comprise the rotations are determined up to their sort order by the requirement that they are not only orthonormal as coefficient vectors but also of covariance zero as linear combinations of the actual measurements case by case. The requirement of zero covariance, algebraically speaking, is just another way to bring in the notion of sums-of-squares (in this context, the sums of squares that stand for variances of the same linear combinations) that parallels this discussion of rotations throughout.^2^

A covariance is a computation that combines *specimens,* not only variables. Its formula is an average of centered crossproducts, $${1\over n}(X_{i1}X_{j1}+X_{i2}X_{j2}+\cdots +X_{in}X_{jn}) - \overline{X_i}~\overline{X_j},$$ and thus appears to beg the questions of what it means to multiply two measurements $$X_i,$$$$X_j$$ on the same specimen and what it means to add these products over specimens; but sometimes that requirement can be circumvented. For quantities in the same units, covariances derive from variances: cov$$(X_i,X_j)=\hbox {var}((X_i+X_j)/2) - \hbox {var} ((X_i-X_j)/2) $$. (In the two expressions after the equals sign here, the $$+$$ and − operators are not regression coefficients but merely instructions about simple arithmetic.) If we can accept the biological reality of a variance as an expected square of a numerical difference of variable values (assuming that *that* makes sense), then a covariance between two quantities in the same units is real or not depending on whether the sum and difference of the corresponding pair of variables can be understood to be biologically real (i.e., properties of the organism) and to have variances that are likewise real properties of the population from which the organism was drawn. And this will be the case only if we can find some process, some gene, some selective gradient that *does* have this pattern of effects on the two scores at the same time. It is far from obvious that any such assumption makes sense. In any event, other covariances will pertain to variables that come with *different* units, for which the preceding identity is meaningless. Furthermore, computed zeroes of covariances are unstable against variations of sample design (choice of taxa, size range, etc.), so composite variates observed to be uncorrelated in one sample will almost surely be correlated in every other sample if the variables being combined submit to any sort of causal reasoning at all.

Then covariances that are exactly zero, which is one aspect of the criterion for our rotation to principal components, would seem to be a property of our scientific rhetoric, not of the organism itself—unless there is some good biological reason to posit the corresponding symmetry. We are thus brought back abruptly to our original paradox: if humerus.length$~\pm ~$aortic.valve.length cannot be taken as biologically meaningful, then neither can the covariance of humerus length by aortic valve length, which is just the expected value of the product of the two deviations from their own sample averages. So the issue of the reality of covariances is effectively the same as the issue of whether linear combinations of independently measured quantities (or of coordinates of independently located landmarks) make sense the way a primary morphometric measurement (an extent—a distance, area, or volume) does. This is a question for a whole team of biologists, perhaps an evo-devo specialist working in tandem with a geneticist. Certainly it does not fall under the remit of the statistician in the room, or the software package that is his avatar.

**Fig. 3:**
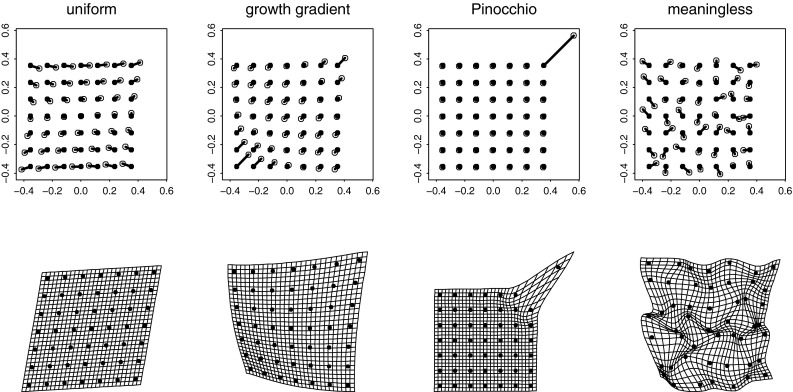
A variety of vectors in shape space. For convenience, each transformation is drawn twice, first as a set of landmark displacements (*filled circle* to *open circle*) in some artificially registered coordinate system and again as a thin-plate spline. Columns, *left* to *right*: a uniform transformation; a growth gradient aligned southwest-to-northeast; a “Pinocchio effect”; a meaningless composite direction in shape space (These latter are the vast majority of available directions; when each of the little vectors is distributed as a circular Gaussian of the same variance, they correspond to samples from the isotropic offset Gaussian shape distribution)

Another way of inspecting the dependence of GMM on rotations is to carefully examine the a-priori symmetry claim that “all directions [linear combinations subject to a geometric normalizing factor] are equivalently plausible a priori.” But this is an absurd position to hold when the subject is patterns of change in landmark configurations, the central concern of GMM. Figure 3 shows a collection of different patterns that the axiom would have us accept as equivalently plausible on this model (they have the same Procrustes length). But biologically they are nothing of the sort. One can imagine a claim that column 1 is detecting the consequence of some biomechanical cause uniformly distributed, or that column 2 is a classic morphogenetic growth gradient parameterized by the relation to some embryonic field along the obvious direction. And a pattern like that in column 3 might be interpreted as a “Pinocchio effect,” the variability of one single landmark irrespective of any phenomena affecting its neighbors. But what are we to do with a linear combination like the one shown in column 4? (There are many more examples of this sort of patternless grid in Bookstein 2015a, b.) We have no scientific access to biological processes that produce this kind of totally decorrelated “pattern.” It follows that however we represent our domain of possible linear combinations, examples like this one must be deprecated. But what, exactly, do we mean by saying an example is “like” one of these? It turns out to be the same geometrical formula (a sum of squares) that we have already agreed has gone unjustified thus far in the application to landmark locations specimen by specimen.

Because the possible patterns that emerge from analyses of rotations of shape variables, such as those in Fig. 3, are intrinsically different in their biological import, it follows that rotationally independent metrics are inappropriate for reporting findings that involve aspects of shape spaces. This caution applies with particular force to the *RV-coefficient* (Robert and Escoufier 1976) sometimes used for comparisons of shape phenomena to exogenous measurement domains, or to more up-to-date modifications such as that of Smilde et al. (2009). Morphometrics needs not some summary assessment of “all the dimensions” of a multimodal comparison but the explicit biological interpretation of eigenvectors or other partial descriptors one by one. If *X* is a matrix of shape variables (such as the shape coordinates of the next section) and *Y* is a matrix of some other measurements on the same specimens, then the *RV* is the sum of the squares of the elements of the matrix $S_{XY}$—the covariances of each *X* with each *Y*—after a peculiar normalization of each matrix separately. Irrespective of the contents of *Y* (which may well be another set of shape measures), then, because the matrix *X* does not encode the spatial adjacencies of the underlying landmark configuration, neither can the covariances of the columns of *X* with the columns of *Y*. Regardless of the details of those normalizations, the procedure makes no sense as biology, inasmuch as many of the numerous patterns over which we are summing could well be nonsensical. If there is some prior reason to consider patterns of covariances as informative, one should be examining the structure of those cross-covariances $S_{XY}$ by a singular-value decomposition of its own, followed by interpretation of individual eigenvectors. “The complete set” has no biological reality. Appendix 1 presents an expansion of this argument that includes a diagram relating this *RV* formula to our usual geometric understanding of covariance structures in the natural sciences. From the proper understanding of the RV coefficient it will follow, the Appendix claims, that it is valueless in most organismal applications.

### Procrustes Distance, Procrustes Coordinates

Let us agree that the first task of the geometric morphometrician is to collect all of the landmark configurations in one data set, so that their coordinate configurations may be treated as causes or effects of other biologically relevant measurements. The commonest way of proceeding with this task is by a *Procrustes analysis.* We now have enough machinery in place to understand what the symmetries of the Procrustes algorithm are and how important it is to be able to break them.

An algebraic version of this task is easiest to set down when we limit ourselves to the realm of “small variations.” Consider each set of measured Cartesian coordinates as if it derived from some common mean form by variation of every coordinate at the same time in the vicinity of its own mean. For convenience I will annotate the situation for a two-dimensional data set of *k* landmarks, thus, 2*k* coordinates (the same as the eventual count of shape coordinates). Write each landmark configuration as a 2*k*-vector distributed around some mean form $$\mu $$. It makes our notation easier if we standardize $$\mu $$ as a vector of the form $$(x_1,y_1,x_2,y_2,\ldots ,x_k,y_k)$$ with $$\Sigma x_i = \Sigma y_i = \Sigma x_iy_i = 0,$$$$ \Sigma \left( x_i^2+y_i^2\right) = 1$$ (meaning: $$\mu $$ is centered, its Centroid Size is 1, and it has been rotated to principal axes horizontal and vertical).

Then it can be shown that the standard *Generalized Procrustes Algorithm* of Gower (1975), which everybody uses for their Procrustes shape coordinates, replaces every 2*k*-vector *C* of data by a new vector very nearly equal to $C - \Sigma _{i=1}^4 J_i^t(J_i C)$ where each $J_i$ is the *i*th row of the matrix

$$\begin{aligned} J = \left( \begin{array}{ccccccc} 1/\sqrt{k}&{}0&{}1/\sqrt{k}&{}0&{}\ldots &{} 1/\sqrt{k} &{} 0 \\ 0&{}1/\sqrt{k}&{}0&{}1/\sqrt{k}&{}\ldots &{} 0 &{} 1/\sqrt{k}\\ -y_1 &{} x_1 &{} -y_2 &{} x_2 &{} \ldots &{} -y_k &{} x_k \\ x_1 &{} y_1 &{} x_2 &{} y_2 &{} \ldots &{} x_k &{} y_k \end{array}\right) , \quad 4 \times 2k. \end{aligned}$$

The first two rows of *J* center the distribution at a common mean of (0, 0). The third row approximately standardizes rotation (by zeroing out torque against the average), and the fourth row approximately standardizes Centroid Size, which is the sum of squared distances of the landmarks from that new centroid.^3^ These four rows are orthogonal in their own geometry of sums of crossproducts, and each has length 1 as a vector. The rotation referred to here is not the sort of rotation with which section “Rotations, Especially Their Basis in Covariance Structures” was concerned. Those were the rotations that could interchange or reproject shape coordinates nearly *ad libitum.* The rotations approximately implemented via the third row of *J* are just the rotations of the digitizing plane as a rigid body, the multiplication of all the shape coordinates by a matrix

$$\begin{aligned} \scriptstyle \left( \begin{array}{cccc} \left( \begin{array}{cc}\cos ~\theta &{}-\sin ~\theta \\ \sin ~\theta &{}\cos ~\theta \end{array}\right) &{} O&{}\ldots &{}O\\ O&{}\left( \begin{array}{cc}\cos ~\theta &{}-\sin ~\theta \\ \sin ~\theta &{}\cos ~\theta \end{array}\right) &{} \ldots &{}O\\ O&{}O&{}\ldots &{}O\\ O&{}O&{}\ldots &{} \left( \begin{array}{cc}\cos ~\theta &{}-\sin ~\theta \\ \sin ~\theta &{}\cos ~\theta \end{array}\right) \end{array}\right) \end{aligned}$$

where *O* is a little $2\times 2$ matrix of zeroes.

To modify *C* by subtracting $\Sigma _{i=1}^4 J_i^t(J_i C)$ is to *project out* the four dimensions expressed in the rows of *J*. That geometry could also serve as the geometry of one morphometric analysis *if all the original Cartesian coordinates were uncorrelated and had the same variance*—if the original coordinate data had been generated as samples from $N(\mu ,\sigma ^2I_{2k})$. This is the so-called *offset isotropic Mardia–Dryden distribution*; for the corresponding probability distribution of shapes, see Dryden and Mardia (1998), Section 6.6.2. Projection leaves distances unchanged that lie in the space orthogonal to all the directions that were projected out. Hence the common didactic simplification that “Procrustes distance is the minimum Euclidean distance between two landmark sets over variations of scale, position, and orientation.” In this *J*-matrix approximation we don’t have to minimize over those nuisance parameters, but just project them out—the distances are, so to speak, minimized automatically. It follows, also, that principal components of Procrustes shape coordinates serve as one set of *principal coordinates* of Procrustes distance, Bookstein (2014), Section 6.5.1.

But no actual morphometric data set is ever distributed with as much symmetry as that $\sigma ^2I_{2k}$ that was just invoked. Whereas the first two rows of *J* normally correspond to nothing measureable outside the digitizing lab, aspects of biological *size* and biological *orientation,* the other two rows, typically are correlated, often *highly* correlated, with the remaining information, the shape coordinates. The shape coordinates emerging from the project-out-*J* algorithm have very nearly the minimum sum-of-squares around their mean of any set of coordinates that stand for “the orbits of the observed data under the action of the similarity group”—all the possible positions, sizes, and orientations we might have assigned them for purposes of this statistical analysis—but the symmetries of that sum of squares are the logical equivalent of the symmetries of the multivariate Gaussian model $N(\mu ,\sigma ^2I_{2k})$ justifying the entries of *J*, and hence are just as arbitrary as *J*’s rows themselves were.

We noted in section “Matrices” that the geometrical structure of a set of Procrustes shape coordinates—some pairs of variables, but not others, pertain to the same landmark point; some pairs but not others represent coordinates aligned in the same direction—is not coded anywhere in the conventional matrix of their values. Breaking this particular symmetry requires careful attention to the specific geometry of a covariance between the various types of these pairs. Furthermore, there is an interaction between the representations of covariance and the *J*-matrix that was projected out in order to pass from Cartesian to Procrustes coordinates in the first place. These concerns, while important, would distract us from the main business of this section; they have been collected for separate consideration in Appendix 2.

The formulation of the *J*-matrix helps us understand why the Procrustes toolkit is particularly incongruent with biology for data sets that incorporate semilandmarks (recall Fig. 2) as well as landmarks. The spacing of semilandmarks is arbitrary, and likewise their weighting in any overall geometric formulation. And the more closely they are spaced, the higher the correlation of their Cartesian coordinates. Among the standard methods available as of the date I am writing this, the only approach that seems robust against this particularly arbitrary choice of parametrization is the method of deflation reviewed in section “Deflated Procrustes Analysis”. Spacing of semilandmarks is a technicality, but allometry, the dependence of shape on size, is a biological fact. The size standardization implicit in the *J*-matrix is the differential of Centroid Size, thus, a *geometric* size. There are still very likely to be correlations of actual biometrical size, *considered as an exogenous biological measurement*, with the shape coordinates (although it makes no sense to project out yet another size variable; instead one would *replace* the fourth row of *J* by some better version, or even omit it entirely as described in the next paragraph). Similarly, the Procrustes shape coordinates may very well show a dependence on orientation of the specimen, likewise considered as an exogenous biological measurement: a consideration that, though perhaps encountered only rarely in systematics, might well arise in a biomechanical study of locomotion. In that context one would delete or replace the third row of *J* for the same reason. Even the first two rows, the centering, might be replaced by a weighted scheme if the landmarks were closely enough spaced for each to represent a patch of tissue; then we could center by approximate area rather than treating the landmarks as identical point masses. Or, in the context of an analysis of gait, we might wish to center the horizontal domain, but not the vertical, so as to preserve the information about potential energy as part of the analysis.

**Fig. 4:**
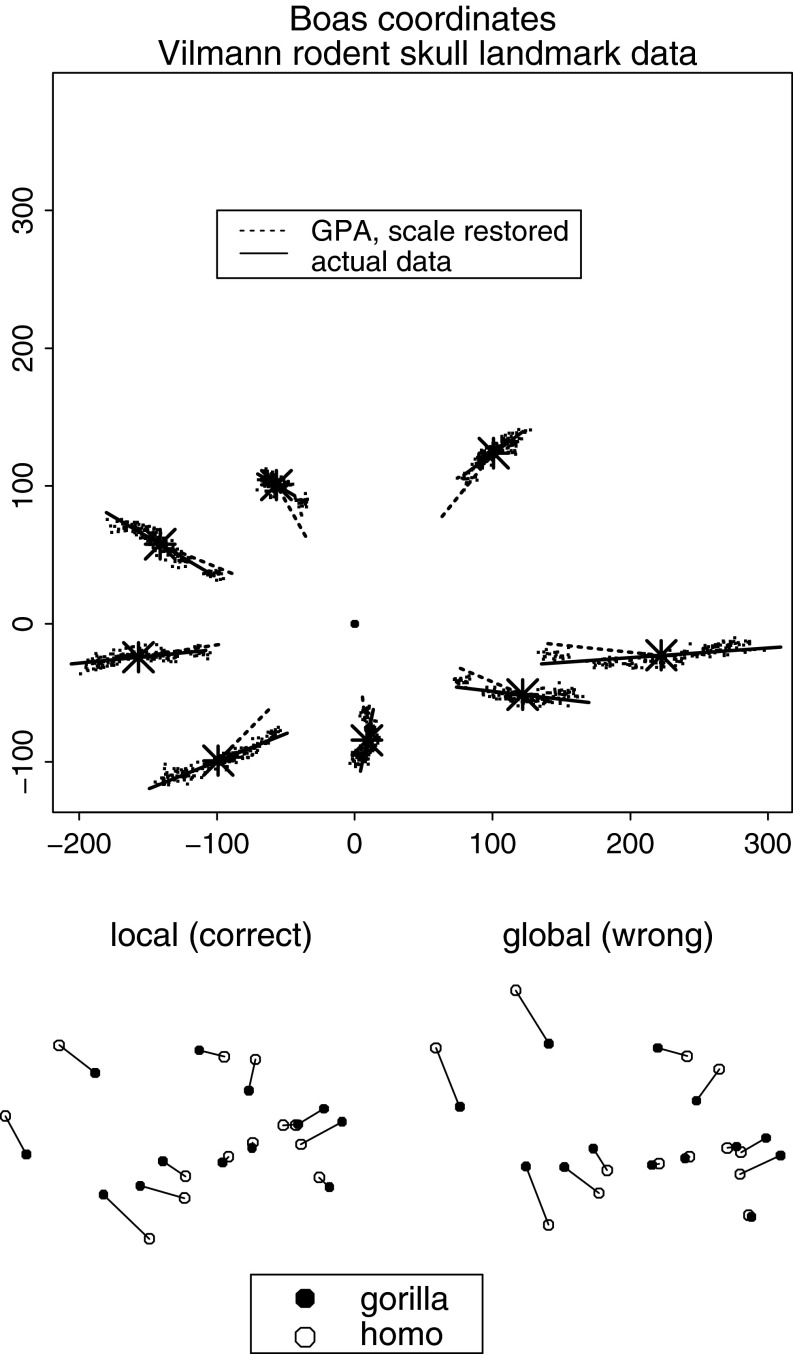
Two kinds of problems with the *J*-matrix. (*above*) Boas coordinates for the Vilmann rodent brain data set. To the extent the little regression vectors on centroid size (the measure of scale divided out in the Procrustes procedure) are not along the directions out of the centroid (the *large filled dot*) and proportional to displacement, the Procrustes procedure has misregistered these data. *Big star symbols* landmark mean locations after centering and rotation (the first three rows of the matrix *J*). *Solid heavy lines* regression predictions for two standard deviations of centroid size in either direction from its mean. *Dashed heavy lines* segments from the means about a third of the way back to the centroid. (*below*) In the method of contrasts, it makes quite a bit of difference whether the projection used to quantify contrast by contrast is based on the grand mean or instead on the pair of forms involved in the specific contrast. The relation between the 13-landmark configurations of *Gorilla* and *Homo* from the Marcus data set to be discussed in section “Modifying a Comparative Analysis of Mammalian Skulls” is clearly different depending on which reference mean is used to construct the projection matrix *J*. *Left*, the correct (local) computation. *Right*, the less thoughtful alternative based on the “mammalian archetype” in Fig. 8, wrongly indicating a much greater relative expansion of the braincase in *Homo.*

Thus the Procrustes superposition itself, which supplies all the shape coordinates that drive the subsequent principal component computations of GMM, its regressions, PLS analyses, etc., encapsulates symmetries that often the biologist would do well to break. A good way to show the problem is by use of the coordinates recommended by Boas (1905), an astonishingly early date. These *Boas coordinates* (a name coined by Joe Felsenstein) are just the shape coordinates of a Procrustes-like procedure that foregoes the scaling step. The upper panel of Fig. 4 shows these coordinates for the familiar Vilmann rodent skull data set, eight landmarks observed in 21 animals at eight ages. (For a listing of the data, see Appendix A.4.5 of Bookstein 1991.) If regressions (here the heavy solid lines) of each landmark position on the summed squared central moment of the configuration are not along the direction of the lines toward the centroid (here the heavy dashed lines), the third and fourth rows of *J* have not been set optimally. In this context the role of an initial Procrustes analysis would be to estimate the correct third and fourth rows of *J*, followed by a recomputation that used these vectors to break the original symmetry. In this example it appears that the standard matrix *J* particularly overweights the landmarks Bregma and Lambda of the anterior cranial roof—their dependence on Centroid Size seems much weaker than would be proportional to their distance from the centroid. The heavy solid lines here show the geometric structure of this octagon’s growth allometry better than the corresponding analysis of the shape coordinates themselves, Bookstein (2014), Figure 7.5 or 7.6.

There is a more subtle problem with the conventional Procrustes superposition when it is applied to a data set of relatively broad shape range. The elements of rows 3 and 4 of the *J*-matrix are the normalized coordinates of the mean shape for whatever sample was being analyzed. Interpretations of the resulting shape coordinates, however, might highlight particular pairs of forms; and, properly speaking, any such comparison should be referred to a *J*-matrix of its own. In the method of contrasts to be introduced in section “The Comparative Method for Analysis of Contrasts Across a Phylogeny”, for example, there will be a different subsample of the data for each contrast of the rotated basis, and thus there should have been a different *J*-matrix for each contrast. The lower panel of Fig. 4 shows the effect of this option for one of the contrasts generated in the Marcus data set of 55 taxa of mammal skulls we will eventually analyze in section “Modifying a Comparative Analysis of Mammalian Skulls”. The analysis on the right, based (inappropriately) on registration to the grand mean form, quite noticeably exaggerates the difference between the forms of *Homo* and *Gorilla* skulls by comparison to the version at left, registered on the average of *Homo* and *Gorilla* only. The region where the registrations most disagree happens also to be the region where the shapes differ most, as a pair, from the putative ancestral form—the most interesting feature of the whole analysis and, we shall see, the reason that conventional principal component 1 of the full 55-taxon data set is worthless as a quantification of variation in any wider context.

The *Procrustes distance* between any pair of specimens is approximately equal to the sum of squares of interspecimen differences of all the coordinates after the four rows of *J* are projected out. This sum of squares likewise is afflicted by all the symmetries of the usual ostensibly isotropic Gaussian distribution, and so usually does not correspond to any biologically plausible version of a meaningful disparity between shapes. Centroid Size is geometrically orthogonal to all the components of Procrustes distance in this context. Its formula is likewise a sum of squares, and its orthogonality to the shape coordinates (and to rotation) is a geometric orthogonality, usually not a statistical noncorrelation. The Procrustes coordinates, properly construed, can serve only as joint causes or effects of form.^4^ They do not constitute a uniquely appropriate quantitative representation of landmark shape, but only one selection from a very rich parametric range of choices. In the lower panel of Fig. 4, the Procrustes distance between the *Gorilla* and *Homo* 13-gons is 0.643, but the wrong analysis (at right) yields 0.710 instead, and owing to the very short divergence time between these two genera it is assigned an unfortunately great weight in some versions of the ensuing multivariate analysis. We will see the consequences of this in section “Modifying a Comparative Analysis of Mammalian Skulls”.

## Some Alternative Methods

For many years, the roster of concerns sketched in section “Four Ubiquitous Problems” has proven a professional challenge to the builders of morphometric tools. How do we build methods that accommodate the circumvention of conventional axioms when the unreality of such axioms is obvious in advance? Here are some of the more important responses to that challenge.

### Relative Eigenanalysis

**Fig. 5:**
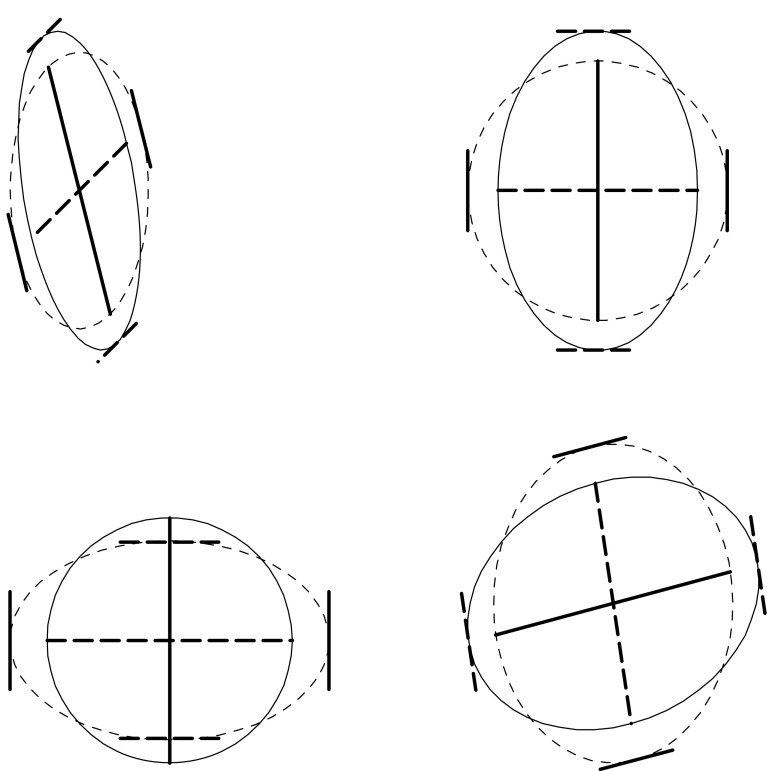
The two basic ideas of relative eigenanalysis. (*upper left*) For any two ellipses, such as covariance matrices of a pair of measurements in two groups, the relative eigenvectors are the directions that are *conjugate* in both of the ellipses at the same time. (A pair of diameters of an ellipse is conjugate if the tangents at the endpoints of each diagonal are parallel to the other diagonal.) (*upper right*, *lower left*) The relative eigenvectors can be computed as well as the axes of either ellipse when the other is linearly transformed into a circle. (For a *circle*, all pairs of perpendicular diameters are conjugate.) (*lower right*) Yet another linear transformation of the same pair of ellipses. The natural distance function between two ellipses is the same in all these panels: the square root of the sum of the squares of the logarithms of the ratios of length between the paired diameters (the relative eigenvectors) of the two ellipses. Here that distance is 0.344

The germ of this idea was planted well before the end of the nineteenth century in the literature of continuum mechanics, specifically, the modeling of material strain as a function of load in settings where the physical stress-strain tensor is not isotropic. The general idea is that the computation of a set of principal components is an algorithm with two arguments: not only the the covariance structure or other symmetric matrix under examination, but also the ancillary square matrix defining what it means to be “orthonormal.” The usual principal components computation is a relative eigenanalysis with respect to the identity matrix (all zeroes except for 1’s down the diagonal), a matrix that has nothing much to do with any biological context. By liberating that second matrix so that it might likewise be informative, the technique of relative eigenanalysis offers a startling enrichment of our usual pattern search engines for situations characterized by prior biological knowledge to which an identity matrix is irrelevant. For instance, relative eigenanalyses are unaffected by diversity of the units in which variables are measured (whereas the usual PC computation would change drastically if the identity matrix were replaced by one with diagonal entries varying substantially around 1.0 in any realistic way). The Vienna theoretical biologist Philipp Mitteroecker has been particularly interested in this extended technology (see Mitteroecker and Bookstein 2009; Bookstein and Mitteroecker 2014). The search for dimensions of shape that are relatively most or least variable in one sample vis-à-vis another, for instance, is resolved by an explicit relative eigenanalysis; so is the generation of deflated Procrustes principal components to be sketched in the next subsection (Fig. 5).

Corresponding to any relative eigenanalysis between matrices of full rank, there is a distance metric for the net dissimilarity of the two matrices being compared. The squared distance is $\sum \log ^2\lambda _i$ where the $\lambda$’s are the relative eigenvalues of either matrix with respect to the other. From the biological point of view, this metric has the happy property that the rescaling of any single factor of the covariance structure has the effect on the resulting covariance geometry of extension along a straight line—successive inflations of the factor add their lengths on a log scale (rather like a slide rule). Also, there is a deep connection between relative eigenanalysis and the main statistical foundation of our multivariate computations, the Wishart distribution of sampling variation of covariance matrices (Wishart 1928): multivariate Gaussian variation around a mean covariance structure is spherical in this distance measure.

Hence, whenever there is a “natural” reference covariance structure in any biological context, we can use it to render our principal components a great deal more comprehensible than if we relied solely on the nonbiological geometry of sums of squares.

### Deflated Procrustes Analysis

This maneuver, while remarkably recent in its formal appearance (Bookstein 2015a, b), derives ultimately from notions of self-similarity that date back to the initial findings about Brownian motion at the turn of the twentieth century. Real Brownian motion, as first demonstrated by the physicist Perrin (1913/1923), is self-similar, the same shape, statistically speaking, at every scale (temporal window) of observation. It has been argued (Nei 2007) that Brownian motion of the phenotype corresponds closely enough to a mechanism of selectively neutral mutational processes that it can often be considered the correct reference model against which to cast claims of evolutionary patterns. (This phrasing is to be taken somewhat elliptically. At larger time scales, neutral drift is not distinguishable from directional selection varying in a suitable joint distribution of direction and magnitude. Also, the variance induced by these diffusive processes will probably vary over directions in our morphometric space—the corresponding Brownian motion would be “colored,” not white. See Felsenstein and Bookstein 2016.)

The equivalent in GMM of the temporal windowing criterion is a spatial one: a shape change phenomenon that is the same shape distribution, statistically speaking, in neighborhoods of every size, position, or orientation. (The claim is not that shape is like position of a particle, but that the ways we allow our focus to move and change scale in studies of a diffusing particle are analogous to the ways we allow our focus to move and change scale in descriptions of shape comparisons.) As modified for application to landmark data, this is the requirement that the reference distribution against which we judge pattern claims needs to offer the same apparent signal for every shape phenomenon at every scale consistent with the mean landmark configuration. This is the equivalent for morphometrics of E. T. Jaynes’s characterization of the familiar Gaussian distribution as the proper representation of “total ignorance” of the information in the statistical distribution of a scalar about which we know only the mean and the variance.

**Fig. 6:**
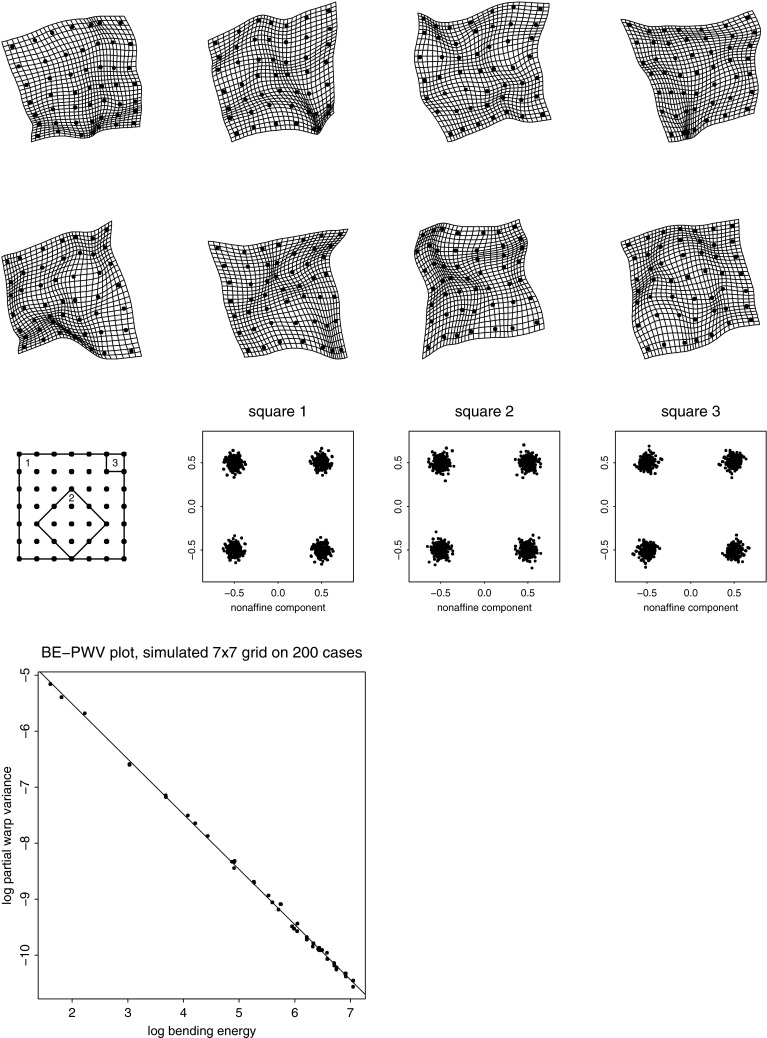
Deflated Procrustes analysis. (*upper two rows*) Eight instances from a sample of 200 from the *deflated Procrustes distribution* (Bookstein 2015a, b) on a $7\times 7$ grid. (*third row*) Nonuniform component of the shape distribution for three diverse squares as indicated in the key figure, *far left*. The theorem says these distributions must be identical. Notice how much less regionalized these grids are than the rightmost grid in Fig. 3. (*bottom panel*) For this distribution, the scatter of partial warp variance against partial warp bending energy, its *BE–PWV plot*, has a slope of $-1$. For this sample the computed slope is $-0.986.$ Empirical data sets often show slopes that differ from $-1$; we will examine one of these in section “Extending the New Morphometric Models of Disorder to a Multiscale Regime: An Example from Entomology”

For any mean landmark configuration there exists such a distribution. The upper half of Fig. 6 shows a sample of eight of these transformations for a scheme of “landmarks” forming a $7\times 7$ grid. The statistical shape distribution of the nonuniform component of any square you might construct from the points of this grid—any size, any position, any orientation—is exactly the same.^5^ This is shown in the middle panel for three squares selected as shown in the guide figure. To put the matter most provocatively, whenever this distribution fits a data set it follows that every single feature that leaps to the eye in an individual grid is as meaningless as the pattern of peaks and slopes of a random walk: those creases or centers of expansion could have been anywhere else, at any scale—they do not bear interpretation. You may be more than astonished—perhaps the American idiom “flabbergasted” and the British “gobsmacked” are the only thesaurus entries energetic enough for this purpose—that such a distribution exists at all, let alone that it can be simulated easily by simple software. In fact, if we remove the uniform component of shape variation from our descriptor space (i.e., the component that is “at infinite scale,” the same everywhere), then we produce a self-similar shape distribution from any isotropic Mardia–Dryden distribution (the shapes of points distributed around their means as circles or spheres all of the same standard deviation in every direction, Dryden and Mardia 1998) as the vectors of scores generated by a relative eigenanalysis of the corresponding shape coordinates with respect to the bending energy matrix of the thin-plate spline (in 2D) or its square (in 3D). One consequence of this scaling is that the formalism is robust against changes in the spacing of semilandmarks with respect to landmarks, a fundamental problem in the Procrustes approach reviewed in section “Procrustes Distance, Procrustes Coordinates”. While smooth changes of outline curvature will emerge at large scales in this new analysis, abrupt local changes, such as remodeling of a joint, will be detected as variation at small scale instead. There is no space to review these helpful features here; for explanations, see Bookstein (2015b).

This result, hinted at 20 years ago in an obscure article on the statistics of the thin-plate spline (Kent and Mardia 1994), makes possible a rigorous multivariate approach to integration centered on visualizations of the patterns by which one claims to have detected it. The partial warps of a landmark configuration (Bookstein 1991, Section 7.5)—eigenvectors of the bending energy of the corresponding thin-plate splines—embody all the information about landmark spacing that lies atop the matrix of Procrustes shape coordinates (and so would have been omitted from the remit of methods like principal component extraction). The proposal is that integration would be best quantified as the regression slope in a *BE–PWV plot,* a log–log plot of partial warp variance against partial warp bending energy. In the bottom panel of Fig. 6 is the corresponding diagram for the $7\times 7$ grid (46 partial warps) based on the full sample of 200 from which the eight exemplars at the top were drawn. The distribution was originally simulated with total isotropic symmetry but then deflated. The slope of the regression line shown is almost precisely $-1.$ Slopes different from $-1$ in either direction convey useful biological information. A claim of integration must therefore be accompanied by evidence that this distribution does *not* apply to the data—that the variance of features falls faster than the inverse of their bending energy. In this circumstance, the deflated RW’s quite effectively convey the aspects of localizable shape variation that are most salient even after adjusting for adjacencies and scaling among the landmarks’ mean locations. The task remains of incorporating the aspect of uniform (affine, hence nonlocalizable) shape variation, which, by virtue of having bending energy zero, cannot be located on our log–log plot without special handling. The approach in Bookstein (2015b) imputes a fictitious scale to the uniform term based on its Procrustes variance; other algorithms should certainly be explored.

### Sewall Wright’s Style of Factor Analysis

Almost exactly 100 years ago, Sewall Wright, analyzing the matrix

$$\begin{aligned} R=\left( \begin{array}{cccccc} 1.000&{}0.584&{}0.615&{}0.601&{}0.570&{}0.600\\ 0.584&{}1.000&{}0.576&{}0.530&{}0.526&{}0.555\\ 0.615&{}0.576&{}1.000&{}0.940&{}0.875&{}0.878\\ 0.601&{}0.530&{}0.940&{}1.000&{}0.877&{}0.886\\ 0.570&{}0.526&{}0.875&{}0.877&{}1.000&{}0.924\\ 0.600&{}0.555&{}0.878&{}0.886&{}0.924&{}1.000\end{array}\right) \end{aligned}$$

of correlations among six size measures (skull length, skull width, humerus length, ulna length, femur length, and tibia length, in that order) for 276 leghorn chickens, noticed that principal components analysis was a remarkably misleading tool for the purpose of biological explanation. (For further comments on this correlation matrix, see Bookstein (2016), Figure 2.31.) All the correlations are positive, and so the first principal component of this matrix, $PC_1=(0.347,0.326,0.443,0.440,0.435,0.440),$ with eigenvalue 4.568, has all direction cosines positive; but every subsequent component is a mixture of positive and negative loadings, as it must be in order to be orthogonal to the first one. For instance, $PC_2=(-0.537,-0.696,0.187,0.251, 0.278,0.226),$ with eigenvalue 0.714, claims to be a contrast between the two skull measures and something like the average of the other four. (This is just an instance of the Perron–Frobenius Theorem recently reviewed for its morphometric implications by Reyment 2013.)

Wright points out, reasonably enough, that process explanations in the biological sciences hardly ever take the form of contrasts like these, and for a factor analysis to be useful it ought to proffer loadings that are sensible guides to biologically distinct processes instead. (This concern will be discussed later under Herbert Simon’s heading of the search for the “nearly decomposable.”) Wright suggested that the subject of the modelling should not be the whole matrix *R* but only its offdiagonal triangles, and that the most useful explanation of the phenomena here would actually be derived from a four-factor decomposition $$R_\mathrm{offdiagonal}\sim g\otimes g + s_1\otimes s_1 + s_2\otimes s_2 + s_3 \otimes s_3$$ where $$g=(0.636,0.583,0.958,0.947,0.914,0.932)$$, $$s_1=(0.468,0.468,0,0,0,0),$$$$s_2=(0,0,0.182,0.182,0,0),$$$$s_3=(0,0,0,0,0.269,0.269)$$, and $$\otimes $$ is the *outer product* that converts a pair of vectors $$(b_i),~(c_j)$$ into the matrix of their elementwise products $$(a_{ij})$$ with $$a_{ij}=b_ic_j$$.

In Wright’s helpful terminology, this is an explanation in terms of one *general size factor,g*, that applies to all six measures, together with three *special factors* each of which applies only to a pair of the original measures: the two skull measures, the two upper limb measures, or the two lower limb measures. The special factors are uncorrelated with the general factor and with each other, and the general factor weighs the skull measures less heavily than the limb measures—the limbs are more correlated with each other than either is with the skull. In this approach, the linear combination with *g* for coefficients serves, after scaling, as the best morphometric estimate available of the *value of the common cause* of those six variables, for comparison with some outside criterion (weight or age, perhaps) claimed to be an expression of the same process. This interpretation of the additive combination—coefficients pertaining to the morphometric variables one by one as *effects* of something—is the counterpart of the version introduced in section “Linear Combinations”, the coefficients as expressing the morphometric variables jointly as a *cause*. (Of course Wright acknowledges there is not really enough information here to identify the actual developmental mode(s) of action of the special factors: Wright 1968:330.)

I have emphasized the role of prior biological knowledge in shaping the broken symmetries of the analyses recommended here. In this setting of multiple length measures the prior knowledge to which Wright’s algorithm has access is the knowledge that these six measures come in three pairs: one pair crossing on the skull, the other two pairs sharing an endpoint (the elbow, for the wing pair; the knee, for the leg pair). Such a decomposition is far more coherent than any principal component analysis can be—it is much more likely that a biological process aligns with the *s*’s, a gene or gene complex for each of the three anatomical compartments, than that some gene system actually accounts for patterns like $PC_2,$ the joint decrease of skull measures along with increase of all the limb measures. (Why should the gene(s) responsible for every principal component after the first be *mandated* to be contrasts? Can’t some pleiotropies—most of them, one could argue—be imagined instead to leave most aspects of an integrated organism unchanged?)

This Wright leghorn example has been discussed at great length in Wright’s own retrospective summaries (e.g., Wright 1968) as well as in the work of others coming later [cf. Bookstein 1985; Marcus in Rohlf et al. (1990)]. If it were not for the numerical quantities, one might think of this procedure as a hierarchical clustering of variables. But those numbers are path coefficients, so the resulting model is indeed an explanatory one. See Mitteroecker and Bookstein (2007).

### Other Modifications in Current Use

#### Regression of One Distance Upon Another

Another approach that circumvents relative eigenanalysis is the replacement of a matrix computation by a matrix of scalar computations. This was the intent of Nathan Mantel’s (1967) original method of matrix-matrix comparisons. The analysis reduces to the estimation of a single scalar, the slope of a regression without intercept of one empirical distance upon another. If we write $d_1,d_2$ for the two distance measurements in question, of course omitting the diagonals of the matrices, then one formula for this slope is just the conventional $\Sigma d_1d_2\big / \Sigma d_1^2.$ Note that the quantity of interest is a regression slope, not a correlation, and that the regression line must go through (0, 0). The method is multivariate only in the sense that the distances driving the regression might be multidimensional summaries, the way that squared distance on a map is the (weighted) sum of squares of change in latitude and change in longitude. Otherwise, the result is not a pattern, but only one single scalar, playing the role of a diffusion constant. The corresponding axioms, then, must deal with the symmetries of that diffusion process per se. For one way of breaking that symmetry, see Bookstein (2007). Extensive modifications of this approach have been explored by Paul Sampson, Peter Guttorp and others to accomodate settings where one of the distances is known to be anisotropic a priori (for instance, migrations across versus along a river, or weather patterns blocked by a mountain range).^6^

#### Domino PLS

This technique was introduced by the Norwegian chemometrician Harald Martens in 2005 by way of diagrams that looked like the playing pieces of the tabletop game called “dominoes.” (The name does *not* refer to the deity in Latin!) It can be thought of as an ad-hoc modification of PLS analysis (the analysis of a crosscovariance matrix $S_{XY}$) to accommodate the type of prior quantitative parameterization described in section “Linear Combinations” (where our context was principal components analysis instead). For each observed block of variables, Domino PLS constructs an auxiliary block expressing the one or more dimensions of prior knowledge in the form of a structured matrix of its own components. Then follows an alternating computation in the spirit of Herman Wold’s original NIPALS algorithm, resulting in a compromise between the optimal cross-block prediction task of the underlying PLS (singular-value decomposition of the matrix $S_{XY}$) and the projection onto the design matrix of the auxiliary block. See Martens and Domino (2005), or the brief exegesis in Bookstein (2014), Section 6.4.3.3.

#### The Comparative Method for Analysis of Contrasts Across a Phylogeny

As an alternative to relative eigenanalysis, section “Relative Eigenanalysis”, one might imagine an approach that used the auxiliary information to construct an expected covariance circumventing at least some of the problems of the standard Procrustes method: in this setting, the presumption that the forms sampled are independent. This is an approach first recommended by Felsenstein (1985) early in the development of his method of “contrasts”—for a review of the history of previous attempts to integrate GMM and phylogenetics, see Section 1.2 of Felsenstein and Bookstein (2016). Contrasts are a rotation of the space of *specimens* to a new orthogonal (but not necessarily uncorrelated) basis of comparisons among individuals or subgroup means corresponding to a presumptive phylogeny. To oversimplify a bit: in the presence of an evolutionary clock, each contrast can be divided by the square root of its duration, whereupon we have a new basis for the space of descriptor vectors (in the GMM application, these are vectors of shape coordinates) the principal components of which are an attempt to reconstruct the domain of neutral selection independent of the accidents of species birth and death, both their directions in morphospace and their locations along the geological time scale. This is the method used for the mammal skull example (section “Modifying a Comparative Analysis of Mammalian Skulls”). For a more detailed explanation, see Felsenstein (2008) or Felsenstein and Bookstein (2016).

## A-Priori Information to Break the Symmetries of GMM

Keeping the critiques of section “Four Ubiquitous Problems” in mind, let us review a range of quantitative insights that most of us would agree ought to be permitted to modify (not just “resemble”) the formulas of our GMM statistical analyses. These claims of causes or effects of form are not to be considered hypotheses exogenous to morphometrics in some sense, so as to be “confirmed” or “disconfirmed” by the morphometric computations. No, they are to be treated as constraints on the morphometric computations themselves—knowledge that must be taken into account in the actual operation of the pattern engines we are exploiting. Our principal components, for instance, need to be computed explicitly in light of those insights regarding, among other things, the regional organization of shape changes. The operators $+$ and − connote explanations of arithmetical combinations across multiple measures only where biology has previously authorized us to do so. Here are some of the contexts where such an authorization might typically be granted.

### The Mean Form

The most important constraint on any GMM analysis is the mean or average form itself. In ordinary multivariate statistics, the mean and the covariance structure are treated as conceptually independent aspects of a population or sample description—in the Gaussian models, indeed, they are statistically independent from first principles. In GMM, by contrast, every aspect of our description of the covariance pattern ought to explicitly accommodate the mean form in its parameters. We saw one example of this dependence already in our discussion of deflated Procrustes analysis, section “Deflated Procrustes Analysis”, and there will be two more instances, semilandmarks and symmetry, in the next paragraphs. The mean form, in other words, is not something to be estimated, or at least not *only* something to be estimated, but also, and principally, the major determinant of our rhetoric for reporting variability. Bookstein (2009) showed the effect of a shift of mean shape on the residuals from the *J*-matrix in the form of its own relative eigenanalysis. The largest and smallest relative eigenvalues of the corresponding pair of null Procrustes distributions are approximately $1\pm \rho$ where $\rho$ is the Procrustes length of that mean shape shift. For realistic shape ranges, as in Fig. 4, this can be quite a large artifact reweighting the space of comparative shape descriptions, as potential factors in the direction common to two means are greatly overweighted in comparison to factors aligned with the direction of their difference. The consequences of one such example are demonstrated in section “Modifying a Comparative Analysis of Mammalian Skulls”.

#### Semilandmarks

One partially hidden role of the mean form that is familiar to most current users of GMM is the way that it is involved in fixing the locations of the *semilandmarks* that sample information from curves in-between the landmarks or surface patches in-between the curves. The formulas for semilandmarks [originally published in Bookstein (1997)] all explicitly involve information about the empirical directions of differential information (tangents to curves or surfaces) at each location being estimated. The current semilandmark formalisms, therefore, already embody one specific example of the symmetry-breaking modifications about which I am speaking: different observed tangent directions lead to different data representations for the same landmark resource. Also, every landmark location affects the location of every semilandmark, whether they share a curve or indeed whether the landmark is located on *any* curve.

#### Bilateral Symmetry

Here in 2016 we already understand quite well how bilateral symmetry is to be imported into GMM. Knowledge of which landmarks are paired and which are unpaired is a fine example of the sort of information that is omitted from the “data matrix” of shape coordinates as reviewed in section “Procrustes Distance, Procrustes Coordinates”. We accommodate that information by *explicitly* dividing our space of phenomena of interest into two subspaces: the “symmetric” and the “asymmetric.” In a typical analysis there are two separate distances or variance components computed, one in each of these subspaces. The subspaces are functions of the pairing of landmarks, and some of the associated GMM operations are explicit functions of the averages after pairing, for instance, the sliding of symmetrically posed semilandmarks reviewed in Bookstein (2014, Section 7.7.6). Note a peculiarity of language in this regard: to *claim* bilateral symmetry is actually to *reject* the standard GMM symmetry that treats all pairs of landmarks as equivalently mutually informative. Bilateral symmetry, in other words, replaces one statistically a-priori symmetry with another more attuned to the actual biological facts of the matter. The subspace for asymmetry, in turn, can be subdivided into several sub-subspaces corresponding to a diversity of biologically disparate processes: bending of the midline, sliding of antimeres along the midline, rotation away from the midline, etc. (Bookstein 2003). All the formulas for these components invoke the coordinates of the mean shape explicitly.

### Allometry

We understand *allometric growth,* the changes in proportion that follow as consequences of changes in size (Gould 1966), better than any other organismal shape phenomenon. In the standard approach to *Procrustes form space* (Mitteroecker et al. 2004, Bookstein 2014, Section 7.4; see also Dryden and Mardia 1998, Chapter 8, who call it “size-and-shape”), size is presumed “just another dimension.” But of course it is not. For one thing, it is more easily observed than any other mensurand (by weight, by net length, or by an obvious proxy like age); for another, it is much more easily intervened upon, as by experimental control of diet or by enforced exercise; for a third, it is already the subject of substantial prior knowledge both as regards ecophenotypic trends (e.g., Bergmann’s Rule) and as regards the variation among species in typical adult body sizes. The current machinery for incorporating size into GMM shares the same Procrustes symmetries that this essay is subjecting to close scrutiny. Part of the new toolkit will need to be a replacement for the current definition of Centroid Size that suspends those Procrustes symmetries in favor of patterns derived from the data at hand. One such approach, relying on a weighted sum of the shape coordinates where the weighting factor for each is the coordinate’s own specific comparative “growth” rate, is currently the subject of computational explorations by Joe Felsenstein and myself (Felsenstein and Bookstein 2016). The ultimate purpose of these innovations is to free discussions of allometry from squabbles over exactly which is the best measure of size should be driving the simplistic regressions driving the standard multivariate approaches. The scare-quotes around the word “growth” above are meant to convey my agreement that these analyses must differ across the fundamentally different kinds of processes (static allometry, growth allometry, evolutionary allometry) that unfortunately share custody of their common term.

### Gravity

Although the three-dimensional space of current GMM treats all directions of a Cartesian triad of coordinates in the same way, the real world does nothing of the sort. Most advanced multicellular life forms show aspects of form aligned with the vertical—they are, so to speak, aware of gravity. Some approaches to morphometrics attempt to accommodate this by fixing a coordinate system—the notorious Frankfort Horizontal of craniometrics, for instance—while other approaches imitate the current methods for bilateral symmetry by splitting the descriptor space into two subspaces, one that includes information about gravitation (e.g., the aspect of gait analysis that concerns the height of the center of mass) and another that does not (kinetic energy of the limbs and trunk, or elastic energy of the ligaments). The division is methodologically complicated inasmuch as height of the center of gravity, and thus potential energy, is linear in the shape coordinates, while kinetic energy is, roughly speaking, quadratic in their rates of change.

### Physiology

The treatment of gravitation as an energy term is one possible locus out of several at which physiological reasoning might be articulated to GMM. Other potential instances come readily to mind: the relative contributions of diaphragm and ribs to changes in lung volume over the human respiratory cycle; the modeling of the Starling equation by aspects of the cardiac ventricular volumes over *that* cycle; the modeling of swimming in water by scaling arguments such as Reynolds number. Because swimming is directional, an otherwise Procrustes-like algorithm can exploit bilateral symmetry to align outline data for a GMM analysis even in the complete absence of landmark points (Bookstein and Ward 2013). The more sophisticated articulation of GMM to mathematical physiology is one of the important areas of future methodological development; I return to this topic in section “Discussion: Solutions Yet to Be Envisioned”.

### Meristic Features

Serial structures like vertebrae have a long history of special treatment in GMM (see, for instance, Slice 2003). A particularly sophisticated example of these approaches, Boisvert et al. (2008), replaces the Procrustes distance formulation *in toto* by an alternative concerned only with the rigid relationships between successive vertebrae. Or the basic scheme of Procrustes coordinates could be preserved and the space still rotated into components for the shape of the vertebrae separately (and their axial trends) versus the relations of each vertebra to its neighbors. This can be seen as a straightforward generalization of the analogous treatment of bilateral symmetry except that the operation of mirroring per se is no longer pertinent.

### Tissue and Image Textures

Gravitation is in the same direction everywhere in the animal body; but some directional information is not so restricted. We are already accustomed to one special case of this concern for tissue texture, the relaxation of semilandmarks along the sharpest boundaries between tissue types or between bony tissue and air. In today’s best anthropometrics this particular broken symmetry is a commonplace. But the technology of this sort of relabeling can be driven a great deal further. In some branches of clinical medical imaging, considerable effort is being invested in the accommodation of texture information to break the symmetries of spatial dimensions by aligning locally instead. Cardiac muscle is known to lie in sheets aligned in different directions; the white matter of the mammalian brain nicely suits descriptors of the directionality of its component neurons as derived from the exquisite new technique of *diffusion imaging*; the image of the retina can be interpreted better by reference to the optic nerve disk that organizes all of its axial symmetries. The retinal coordinate system, in turn, is a special case of a family of descriptors organized on cylindrical instead of Cartesian principles: coordinate systems for tubes such as blood vessels or the intestines, in which one coordinate is linear, one radial, one azimuthal.

### Mechanical Strain

An early response to the complaint in section “Linear Combinations” about the meaninglessness in general of endogenous linear combinations of shape coordinates arose in the course of articulating GMM with the neighboring and even more quantitative field of biomechanics. Responding to challenges over the course of a colloquium on the biomechanics of GMM in 2013, I propounded a formal method for articulating prior knowledge of biomechanical ratios with GMM descriptive statistics. The suggestion is to map the text of any dimensionless biomechanical formula (say, for a physical angle measured on the landmark configuration, or the ratio of a pair of regional measures of extent such as length or linearized area) into an explicit linear combination of shape coordinates that is computed purely algebraically, without any reference to the statistics of the shape coordinates. [The formulas for this conversion had originally been published in Bookstein (1986).] A sample of forms or a taxonomic contrast could then be described by reference to these directions, or a system of principal components could be compared to them.

For the simplest cases, those involving only relative size measures, the computation reduces to diagramming the geometry of each extent in Procrustes space (for a length, equal and opposite vectors at each landmark pointing away from the other; for an area, vectors out of the common centroid proportional to mean distance *from* that centroid), then projecting row 4 of the *J*-matrix from these vectors (in order to accommodate the way they jointly affect the Procrustes superposition and thereby each other’s estimates). The method is explicitly limited to landmark points, points for which each of the Cartesian coordinates is meaningful; it does not extend to the semilandmarks that comprise the bulk of our GMM data sets these days. For the general algebra of this approach, consult the extended exegeses in Bookstein (2015c, 2016).

A second methodological speculation likewise concerns the mutual scaling of form and the sort of response to load that is currently pursued mainly by bioengineering-based finite element models (FEM’s), a computational flow that involves the same data base of information that GMM exploits but treats it very differently. Bookstein (2013a), another paper of mine responding to an explicit challenge from a conference in Vienna in 2010, shows how a concern for actual physical strain energy can bridge the GMM and FEM domains. The approach recommended here is an explicit regression of deformation energy on the GMM descriptor space. The example worked there, a cantilevered rod of varying shape under fixed load, demonstrates that the Procrustes metric of GMM per se has nothing in particular to do with the coefficients in this regression once the geometrical symmetries of GMM have already been broken by the geometry of the load regime itself. The hope is that such computations will extend to physiologically interesting settings like chewing or locomotion. In the setting of load on a cantilever, the two concepts of bending energy, one from GMM and the other from FEM, articulate fairly well inasmuch as both involve integrals of squared second derivatives, but the relative scaling dimensions of the two differ according to specific details of the modeling task at hand. Work is in progress to extend this investigative style to a wide range of other bioengineering contexts, such as the bending of shells (e.g., the cranium), for which analytic approximations exist in the textbooks that obviate any need for the intensive algebraic computations of finite element analysis per se. There is no reason to expect representations of this sort to be linear in the shape coordinates or in any other representation of form.^7^

## Two Evolutionary Examples

The challenge implied by all the preceding suggestions is to build software protocols that explicitly constrain the formulas of GMM by incorporating insights from other branches of biology. To date I have worked one example along these lines and collaborated on another, both, as it happens, from the articulation with bioengineering.

### Extending the New Morphometric Models of Disorder to a Multiscale Regime: An Example from Entomology

The discovery of this example and the sketch of its elaboration are Jim Rohlf’s, to whom I am very grateful for permission to use them here.

**Fig. 7:**
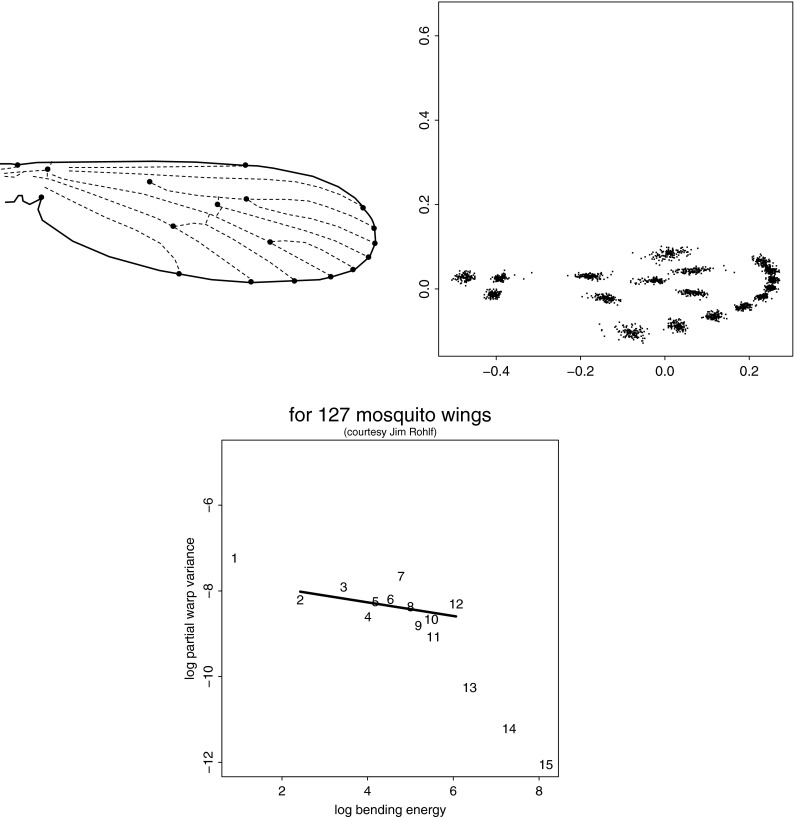
Rohlf’s mixed model for mosquito wings. (*top left*) Template for 18 landmarks of the culicid wing. (Courtesy of Dr. Sonja Windhager, after Rohlf and Slice (1990), Fig. 7.) (*top right*) Procrustes shape coordinate plot for typical forms of 127 species. (*bottom*) The BE–PWV plot, log bending energy against log of partial warp variance (see text)

When extended to the estimate of a self-similarity dimension, the “deflated Procrustes analysis” sketched in section “Deflated Procrustes Analysis” shares one underlying premise with the other covariance-based methods reviewed at section “Rotations, Especially Their Basis in Covariance Structures”: the axiom that a regression slope applies homogeneously across the whole predictor range. In the three examples of Bookstein (2015b), all of which pertain to the mammalian cranium, this assumption seemed compatible with the data. But the very first time a reviewer attempted to extend this range of examples, to a structure that is under very severe biomechanical constraint (the wing) in an invertebrate family (Culicidae—mosquitoes), the axiom failed quite blatantly. See Fig. 7.

In this classic 18-landmark data set, there is a dominant first partial warp (position of the midwing vein landmarks vis-à-vis the overall pattern), a midrange of selfsimilarity dimension zero consistent with an isotropic Procrustes distribution (along the longitudinal axis only), and, finally, a selfsimilar falloff at the finest level of detail. An appropriate interpretation would note that the subset of landmarks that seem to participate in the segment of slope zero in the center of this plot are, by and large, those that show the most elongated scatters of Procrustes coordinates in the upper right figure. These are the landmarks near the middle of the wing, where the vein branching would seem not to have much effect on aerodynamic properties, i.e. to be functionally neutral. At the other end of the plot is a regime of scaling that appears to match Example 2 of Bookstein (2015b) in being a domain of self-similar variation of a curving boundary (in this case, the trailing edge of the wing as airfoil). By explicitly challenging GMM’s standard symmetries we have thus neatly separated the functional from the potentially phylogenetic aspects of this data set.

A corresponding reduced shape distance or shape probability model would partial out the space of partial warp 1, either as one dimension (if it is aligned with a specific two-vector of directions, in this example the direction along the wing’s long axis) or in two dimensions, before applying a spherical Mardia–Dryden model. Effects on this configuration, such as taxonomic differences arising from selection mechanisms, would then be reported as the combination of a directional statistic (change in PW1) and a nondirectional statistic (Procrustes distance in the complementary space). This distance term is spherical in the landmark means but not spherical in the directions of variation around them: the “vertical” (direction of the animal’s motion) is strongly canalized even though positioning along the long wing axis itself is not. This Procrustes distance term, in turn, would be truncated by deflation prior to the end of the PW sequence. Hence the BE–PWV plot confirms the insights a specialist in “life in moving fluids” might have brought to the original data flow: variation of wing landmarks across the Culicidae is very strongly canalized by aerodynamics in two disparate scaling regimes separated by a region that appears to be aerodynamically neutral. The insight, in turn, makes us alter our principal components radically from the set that would otherwise have been supplied by the conventional GMM toolkit.

### Modifying a Comparative Analysis of Mammalian Skulls

Early in his career, in the course of a study of the primate scapula, the great bioengineer Charles Oxnard wrote,

A series of features of the shoulder bones, chosen because of their association with the mechanically meaningful features of the musculature, have been found to vary (a) in association with the known contrasts in locomotion, and (b) in such a way as to render more efficient mechanically the associated muscular structure. Investigation of bony dimensions residual to such a study has shown that they are not highly correlated with primate locomotion but are, in contrast, associated with the commonly accepted taxonomic grouping of the order. (Oxnard 1967, p. 219)

By the turn of this century, he was more assured on the subject. Morphometric descriptions of species differences tend to contrast the functions of various anatomical parts (this is certainly the case in, e.g., discussions of primate or human evolution), whereas discussions of evolutionary relatedness per se tend to combine measurements over many parts of the organism at once and to make particular sense in terms of development. Any competent morphometric analysis must maintain the distinction between these two distinct kinds of explanations. As he summarizes things,

Individual [taxon-specific] studies speak most closely to function and combined studies to evolution. $\ldots$ This thinking relates to a more sophisticated view of what comprises a ‘variable’ or a ‘feature’ or a ‘character’ in morphology. (Oxnard 2000, p. 260)

For a general review of the context in which these views were put forward, see the brief history of principal component methods in Bookstein (2015c).

The biomechanics of a mosquito wing is relatively intuitive, being mainly a matter of wing shape and wing stiffness, and the range of forms in the scatter of Fig. 7 is evidently not too great. Thus we think we know how to manage Oxnard’s distinction in this particular setting. But suppose we are dealing with a greater range of variability and at the same time we are not so certain in advance of the function(s) involved in managing landmark spacing. Then perhaps we can proceed by a study of the scale of the extracted features themselves: for function, at large scale; for evolution, at smaller scales, collectively. The functions need not be identified with individual relative warps but only with the subspace of shape space that they span. (Remember that “relative warps” is the general term for principal components when they arise in a context for which diagrams of warping are appropriate.)

**Fig. 8:**
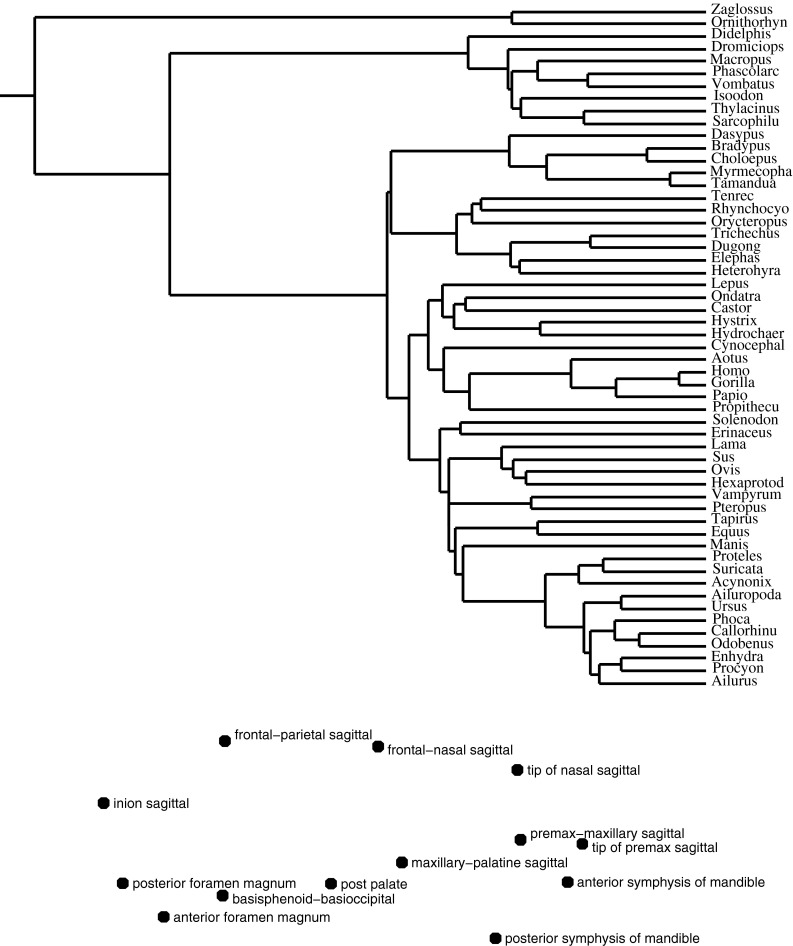
Structure of the Marcus et al. (2000) data set of skull landmarks for 55 taxa of mammals. (*above*) One current phylogeny, courtesy of Joe Felsenstein. The range of the horizontal axis here is about 200 million years. (*below*) Average shape of the midline 13-gons for the 55 representative specimens

Here is an example of that possibility, in an application to part of the landmark data for the main orders of mammals published by Leslie Marcus, Erika Hingst-Zaher, and Hassam Zaher in 2000.^8^ I am told there is fairly general agreement on the reasonable phylogeny shown in Fig. 8, which was downloaded from the Timetree of Life (www.timetree.org) sometime in 2013. The horizontal axis here is scaled by estimated branching date, with a maximum of 176 Myr. The conventional Procrustes mean form of these 55 exemplars is as in the lower panel of the figure. In connection with Fig. 4 I already introduced you to the pathologies of using a mean form so far from the poles of a contrast, in that instance, the contrast of *Gorilla* with *Homo.* That analysis was selected from the full complement of 54 contrasts spanning the 55 13-gons of this exercise. The Procrustes fits here are not quite the standard ones, but replaced the rotation step by a maximum-likelihood procedure (Felsenstein and Bookstein 2016); that detail does not greatly affect the following discussion. The contrasts were computed with individualized *J*-matrices, as recommended in section “Procrustes Distance, Procrustes Coordinates”, and each was scaled by the square-root of equivalent net divergence time according to Joe Felsenstein’s advice in section “The Comparative Method for Analysis of Contrasts Across a Phylogeny.”

**Fig. 9:**
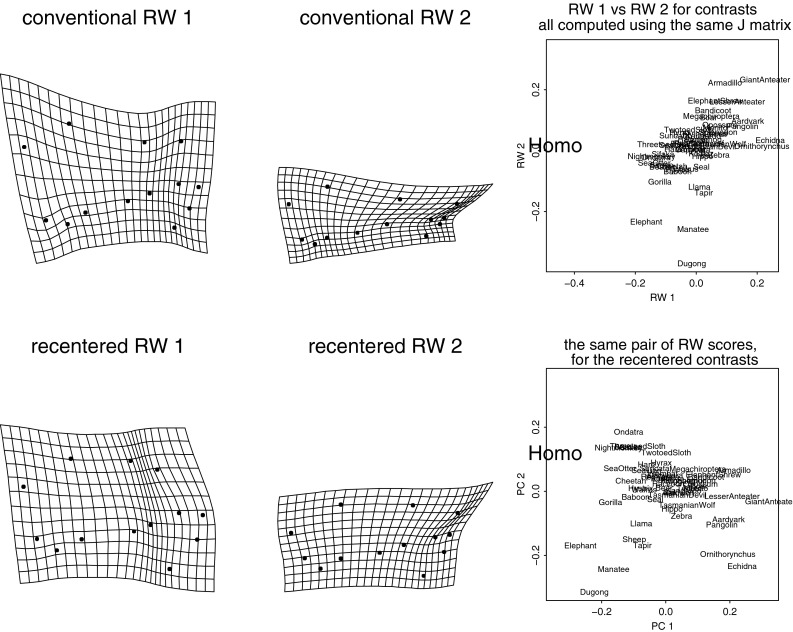
Effect of the recentering on findings from the method of contrasts. Computations are based on time-normalized contrasts from the phylogeny in Fig. 8. Columns, *left* to *right*: relative warps (RW’s) 1 and 2 as thin-plate splines; scatter of scores as reconstructed from contrasts. *Upper row* per the conventional method, which uses a single *J*-matrix for all the contrasts. *Lower row* using a different registration (*J*-matrix) for each contrast. There is clearly an enormous difference in the leverage attributed to *Homo* here

Figure 9 compares the two approaches to the construction of linear combinations suggestive of meaningful dimensions of variation, computed via principal components of the time-normalized Felsenstein contrasts. The figure shows the first two of these dimensions. The left column shows the grid for the first of these dimensions, the center column, for the second; the right column, finally, scatters the reconstructed scores for the individual taxa. It is clear that the recentering procedure (adjustment of *J* in accordance with a different mean value for every contrast) makes a considerable difference for the analysis here. The effect of the aberrant genus *Homo* on the first dimension, in particular, is greatly reduced by the recentering, in keeping with the reduction of its distance to the sister genus *Gorilla* already shown in Fig. 4.

Once *Homo* is deleted from the plots, one of the two Cartesian dimensions is nearly identical between them. The direction (0.865, 0.739) of the conventional analysis correlates 0.998 with the direction (1.123, 0.102) of the recentered analysis. (The extreme forms on both are elephant and giant anteater.) That is the maximal canonical correlation; the minimum one, 0.898, suggests that much of the fine detail of the scatterplot has altered. Even at the coarsest level, notice that that most stable direction has rotated a full $45^\circ$ between the analyses: what is approximately along the first relative warp in the lower row of Fig. 9 nearly bisects the angle between the axes in the upper row. Changes like these, consistent with our discussion of the sensitivity of linear combinations (section “Linear Combinations”) to tiny details of the assignment, render unstable any judgment regarding the relation between morphology and phylogeny across this shape range. For instance, while the forms most distant fron *Homo* in the two righthand scatters remain the echidna, the platypus, and the giant anteater, the ordination on relative warp two is nevertheless remarkably rearranged between top and bottom rows, corresponding to the major change in the balance of anterior and posterior features in its formulation. The *Homo*–*Gorilla* contrast not only is overweighted in the analysis of all 55 taxa but also skews its extracted dimensions quite severely. (And so this comparison also serves as an excellent example of the instability of zero covariances explored in section “Rotations, Especially Their Basis in Covariance Structures”) When principal components are as sensitive to algebraic assumptions as the two pairs shown here, it would be foolhardy to presume that either set, or indeed any set at all, is telling the truth.

**Fig. 10:**
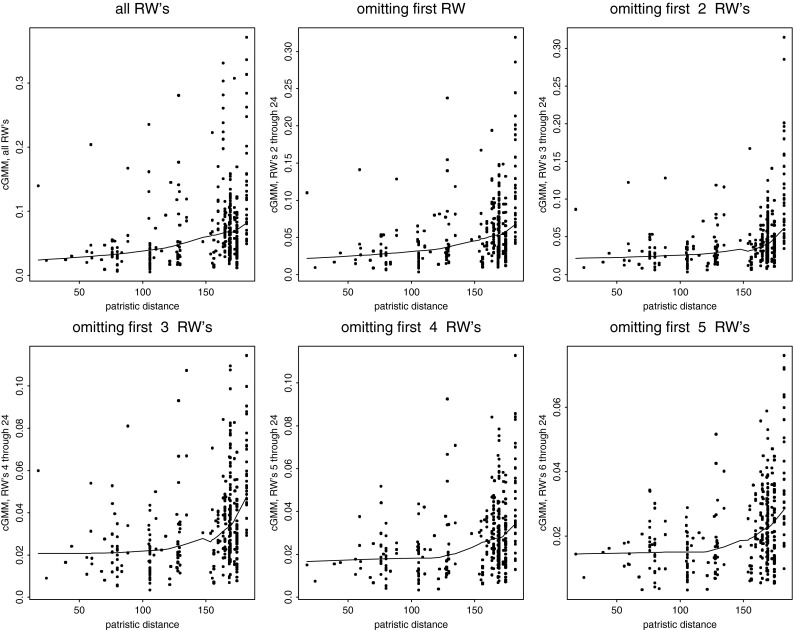
Approaching the neutral model of evolution. Scatters of locally computed Procrustes distance versus patristic distance for the full data set (*upper left*) and then versions omitting the first one, two, $\ldots$, five relative warps (RW’s) of the contrasts. Patristic distance (on the *horizontal*) has been restricted to less than 200 myr. The first scatterplot to show a linear upper border appears to be the fifth (*center bottom panel*), suggesting that there are three dimensions of functional morphology here that need to be partialled out, and also the specific derived features of *Homo,* before we can study anything important about evolution. cGMM: corrected GMM distance computation after recentering the *J*-matrix and (except at *upper left*) projecting out some of the relative warps. *Lines* lowess smooths of the dependence of the ordinate on the abscissa. Notice that the scales of the vertical axis differ from frame to frame (Color figure online)

This has been a discussion of the first pair of relative warps, but possibly, following Oxnard’s hint, we should be looking at the other end of their range: the more numerous warps of lesser, not greater, explained variance. One might guess that methods based on estimates of morphological similarity (of which Procrustes analysis is one) are to be considered reliable and informative only in the absence of functional features, as those are regulated so differently. If we are looking for evidence of neutral evolution, which here would connote a rough proportionality between patristic distance (total divergence time) and morphological distance (as per the Procrustes formula), it must arise as a linearity in the upper margin of a plot from which the functional dimensions, the first few relative warps (such as the two in Fig. 9), have been *removed*—jointly partialled out beforehand. As we see from the lower center panel of Fig. 10, this linearity first appears after the removal of four of these rotated linear combinations. The lines on the plots, which are lowess estimates (locally smoothed regressions), indicate that linear fits of shape distance on patristic distance are unlikely to be meaningful, as already noted in section “Regression of One Distance Upon Another”. For instance, these lines do not seem to pass through (0, 0). Notice also how the slope of the smooth falls to zero below 100 Myr as successive RW’s are partialled out, meanwhile maintaining its acceleration over the last quarter of its range; both features further support Oxnard’s conjecture.

Our example suggests, then, that matches between phylogenetics and morphometrics may be artifacts of methodological choices deeply buried in the Procrustes algebra and geometry. The relation between a phylogeny and a scatter of relative warp scores is far more complex than just projecting a phylogeny over a scatterplot or even a series of scatterplots. A less crassly empirical approach than what is illustrated in Fig. 10 would replace the simplistic suggestion there, the serial discarding of relative warps, by some computation drawing on prior biological knowledge of biological processes, such as the projection of the Procrustes space onto meaningful biomechanical axes prior to any consideration of covariances (Bookstein 2015c) or “association with the musculature” (Oxnard 1967). It would be nice to know, in other words, what the functional interpretation(s) of the space of the second, third, and fourth of these relative warps could be and thereafter whether it makes biological sense to stop the winnowing at four dimensions (three plus the disruptive effect of *Homo*).

## Discussion: Solutions Yet to Be Envisioned

It would be easy to extend the section “A-Priori Information to Break the Symmetries of GMM’s” list of candidates for GMM symmetry-breaking. Clearly some components of our prior knowledge base fit the formalisms of GMM better than others. Growth gradients, for instance, are much easier to model (e.g., as polynomial trends, see Bookstein 1991, 2015b) than the energetics of an elastic vertebral column or an equine foot. An appropriate toolkit would be capable of accepting information in any or all of these channels once translated into a common morphometric language.^9^ The translations will generally consist of parallelization of parameters so as to permit their variation across the organism, thereby breaking the spherical symmetry of the Procrustes method or the principal component method. Section “Some Alternative Methods” reinterpreted a range of current tools from this common perspective, and section “A-Priori Information to Break the Symmetries of GMM” speculated on a variety of tools not yet announced that will accommodate even more of this perfectly unremarkable, intuitively accessible prior knowledge: not constraints on the morphometrics, but an adaptive radiation corresponding to better matches between the algebra or geometry that drives our statistics and the actual biological questions being asked in particular organismal settings. This closing discussion pursues a variety of more general issues associated with all these developments and suggestions.

### What Not to Do

Our examples in Sections “Some Alternative Methods”, “A-Priori Information to Break the Symmetries of GMM” and “Two Evolutionary Examples” have in common the avoidance of several widely encountered bad habits. Owing to these virtues, and in spite of the occasionally esoteric (or at least unfamiliar) mathematical notions sometimes entailed in understanding them, they deserve broader exposure in any context where prior biological knowledge may be presumed to dominate the abstract, tacit symmetry assumptions of the standard multivariate methods. Such a sea-change in our peer-reviewed publications would be good news; likewise in our biology graduate training programs.

#### In Good GMM, Science Is Never Subordinated to Statistics

In all the examples above, the biometrical a-priori completely dominates the logic of the symmetries that incorporate our mathematical inertia. Put another way, *the standard multivariate statistical models do not suit any biological “FAQ page.”* The disorder of representative sampling is highly atypical of scientific ignorance; likewise the disorder of gas molecules, in their Gaussian memorylessness, is entirely incommensurate with actual biological systems, which (cf. Elsasser 1975) are characterized mainly by “nonstructural memory.” Instead we need more new techniques that bring the geometry of landmark spacing (Bookstein 2015b), biomechanics (Bookstein 2015c), bioengineering (Bookstein 2013a), or biophysics (Cook et al. 2011) into the discussion. Hardly any part of organismal biology resembles the random sampling on which Fisher and his disciples based their sampling theories.

#### In Good GMM, Statistical Null Hypotheses Play No Logical Role

This is a longstanding critical theme of statistical praxis ever since the 1930s. Properly understood, GMM is not conducive to any spirit of “null-hypothesis testing.” Its goal is exploratory: pattern description, not decision. Permutation tests do not conduce to excellence in organismal biology, mainly because the corresponding distributions are never encountered as descriptions of processes at the level of the organism. Life isn’t organized as a set of modest perturbations of equilibrium; it is dissipative and far from equilbrium. The features of organisms are correlated at every spatial and temporal scale we have ever examined. The geometric morphometric task is the estimation of path coefficients, not the pretense of yes-no answers.

#### In Good GMM, The Classic Multivariate Symmetries Are Broken Whenever It Is Appropriate to Break Them

Issues of sample exchangeability aside, we have seen that the symmetries of classic multivariate analysis (rotations, sums of squares) hardly ever make biological sense in morphometric applications. As E. T. Jaynes explained (Jaynes and Bretthorst 2003), these symmetries, like the postulate of Gaussian distributions in the first place, are at root expressions of our prior ignorance about the content of the scientific pattern(s) we are investigating: ignorance that is, in most contexts, a hard-won property of the standardization of instruments and experimental designs by which we approach the topic. In biology, we cannot experiment this way. No matter how balanced an ANOVA design, for instance, we cannot induce noncorrelation among the component pathways of any developmental process. In Jaynes’s metaphor, we are *never* ignorant enough in GMM applications to permit the luxury of recourse to these maximally symmetric hypotheses. Variation of landmark locations is not remotely the same, philosophically speaking, as “noise” in some engineering context. Rather, every landmark location is itself accommodating some optimum over a range of possible morphogenetic processes, processes whose constraints are among the a-priori dimensions that this paper is arguing should replace those nugatory original symmetry assumptions.

### What to Do Instead

In place of these classic models of ignorance, GMM may sustain other models much more attuned to the ways in which we actually report the patterns it uncovers. I am particularly enthusiastic nowadays about the toolkit of deflated Procrustes distance sketched in section “Deflated Procrustes Analysis”, exemplified in section “Extending the New Morphometric Models of Disorder to a Multiscale Regime: An Example from Entomology”, and exposited further in Bookstein (2015a, b). The model of self-similar descriptions there corresponds *perfectly,* in my opinion, to the nature of the morphometrician’s prior spatial knowledge before any pattern constraints like those in section “A-Priori Information to Break the Symmetries of GMM” have been applied. At that stage, the statistician has no information whatever about the location, orientation, or scale of the phenomena she is likely to uncover. She is thereby put into the same state of professional unbiasedness as the classic systematist, who looks for keys to classifications under just those same circumstances: a “systematic character” can be *any* discriminant feature, from behavior through net body size through (to take just one example) details of the genitalia (and perhaps much further down the descriptive tree to the level of individual amino acids in a polypeptide’s primary structure). In the future, when principal component analyses appear they should be in the mode that respects *this* prior ignorance, not the ignorance of “rotations of linear combinations,” which is so deeply confounded with the locations and spacing of the landmarks in the mean form.

The concern is the representation of GMM information content not in isolation but as it articulates with all of the other modes of information by which we understand biological form, function, and evolution. Our task as biological scientists remains Socrates’s task as set down in Plato’s dialogue *Phaedrus*: to “cut Nature at the joints.” We know a lot about these joints prior to launching on any particular study (see, for instance, Bookstein 2015d), otherwise we would not have managed to argue successfully for the funding to carry out the study. As Herbert Simon argued in a posthumous publication (Simon 2005), Nature (or at least the part of Nature studied in the natural sciences) tends to be organized hierarchically in “nearly decomposable” systems and subsystems. For organismal biology, those are organs and the joints between them. The version of this advice that is most appropriate for GMM study designs would be the adviso to measure either inside a component or explicitly across a joint, rather than trying to combine these two purposes.

What appears to be missing from the disciplines bounding GMM is, in many domains, a rhetoric for their language of decomposable subsystems that can be translated into the morphometric context. In setting up the appropriate sampling frame for studies like these, it would be appropriate to learn from the concept of “ranges of normal” as reflected in our understanding of human anatomy, for example. But few anatomy atlases actually devote any space to exploring that “range of normal,” the actual manner in which quantitative variation is distinguished from qualitative typologies. [For splendid exceptions, see Anson (1950/1963), or Keats and Anderson (2001). In contrast, Cornelius Rosse’s otherwise superb “foundational model of anatomy” offers no representations at all of variation: see Rosse and Mejino (2003).] The analogous question within GMM itself is the issue of the limits of the deformation model. Oxnard and O’Higgins (2009) have a thoughtful overview of the topic as it pertains to the tendon sheaths of the anthropoid cranium, and many of the current approaches to image analysis of human brains involve experiments in the interplay of continuous versus discrete descriptors of brain form: cortical sulci and gyri, for instance, versus their flattening into a convex prototype, or the analysis of spatial fields by their pixel-by-pixel values versus the decomposition of the same images into “watersheds.”

One example of the corresponding methodology falls under the heading of the Ontology of Physics in Biology (OPB) published by Dan Cook and colleagues a few years ago (Cook et al. 2011, 2013). Cook systematically surveyed the domain of biophysics for the terms that appeared to function in common across examples—terms like mass, energy, action, flux, force—and has built a corresponding computer-accessible glossary that makes sense, for instance, of the differences between fields and their integrals, or boundaries and the flows across them. Originally funded to systematize the literature of cardiovascular physiology, the OPB’s terminology is intended to form the underlay for a systematic extension of notions of spatial occupancy from morphometrics (in the broad sense) over into all of the biological sciences that involve studies of energy and its transformations. Simon’s principles are certainly honored as well in the division of the underlying anatomy into its component parts. For the circulatory system, these would be (the Latin equivalents of) the heart and its chambers, valves, individual blood vessels, and, most important, the flowing blood that occupies all of the spaces within these compartments and bears the oxygen and glucose that embody the difference between life and death. Remarkably enough, the OPB has no role for landmarks in its semantics. It is worth pondering that discrete points do not appear to be of much use for specifying the nature of biological control of physiological systems.

At a much smaller spatial scale, computational settings that would otherwise involve the GMM of components of molecules, for instance, are governed not by Procrustes distance but by explicit formulas that calibrate the *real* configurational energy of particular molecular configurations, energies that likely control the corresponding Brownian motions. See, for instance, Theobald and Wuttke (2008) or Hamelryck et al. (2015). Theobald has publicized a computational framework, the *Theseus* software package, for analyses quite unrelated to the Procrustes versions of shape coordinates of atom positions in proteins.

Considerations like these evoke the more salient question as to whether the GMM focus on anatomical landmarks has much to offer biology beyond its historical role in systematics in general and such fields as anthropometrics and animal husbandry in particular. We can agree that landmarks are often helpful in systematics and in classification in biology and in medicine [for one unusual domain of application, see Bookstein and Kowell (2010)], but otherwise, when *do* landmarks make sense in evo-devo biology, or functional biology? One way of calling the question in this domain is to ask when, if ever, landmark locations are the correct formalism for describing effects *on* form or effects *of* form. The current trend in medical image analysis, for instance, goes in a somewhat different direction: recourse to coarsely registered eigenimages for solids, for instance [see, e.g., the technique of “voxel-based morphometry,” VBM, which I have considered vis-à-vis GMM in Bookstein (2001)], or the exploitation of networks of characteristic gray-scale features for surface images like faces (Taigman et al. 2014).

In contrast, the Icelandic-Canadian geneticist Benedikt Hallgrimsson has shown us several pretty examples of the relevance of landmark-based GMM to studies of knockout models for human birth defects. Such work may well ultimately be considered a *locus classicus* of the use of landmark data as dependent variables in studies designed to assess the information in landmarks as calibrations of effects. In Hallgrimsson’s work [see, for instance, Hallgrimsson et al. (2009)], landmarks serve as hints about genetic effects on potentially measureable extents (lengths, volumes) that, in turn, are known to be outside the normal range in studies of particular human genotypes. In a compromise of another sort, the current technologies of endoscopy reduce tubular structures (bronchi, intestines) to their cylindrical coordinate systems, as already mentioned in section “Tissue and Image Textures”. We can thus keep track of one spatial coordinate, the distance we have come, without being able to embed it in any sort of more extended 3D system.

A final suggestion along these lines would have us change our focus from the subject of random variables to the broader topic of random matrices. There we find an important model of “total disorder” that is quite different from the kind of pattern our current covariance-based tools detect effectively. Imagine a matrix of specimen-by-specimen distances (dissimilarities) that, beyond any low-rank pattern of determination by factors, incorporates independent, identically distributed additive random noise in every symmetrically placed pair of off-diagonal cells. This model, which is quite realistic in certain applications in the physical sciences, can be detected with the aid of a histogram of the eigenvalues of the corresponding eigenvectors. According to the celebrated *Wigner Semicircle Theorem,* that histogram should take the specific form of a simple semicircle in all regions of the spectrum distinct from the fixed effects of those factors. Protocols for the detection of such disorder would lead to insights into the fine structure of morphometric variations at least as important as the protocols for reviewing the large-scale patterns that are the domain of today’s thin-plate-spline toolkit.

In my view the lack of speculation on these and related matters—the limits of the landmark formalism and thereby the pattern analyses it sustains—is one of the main lacunas accounting for the relatively low profile of geometric morphometrics across the biological sciences today in comparison to the only slightly older techniques of comparisons among group averages that continue to dominate the journals in most application domains. Many of us have been struck, for instance, by the relative nonpenetration of GMM into such eukaryotic kingdoms as botany or protistics. I may have persuaded you, or at least opened your mind to the possibility, that this is our own fault—that Procrustes distance is not a particularly realistic formula for most investigations into organismal biology: it is just too symmetrical. The deeper issue is whether the notion of a “landmark point” still makes sense here in the twenty-first century the way it did for Rudolf Martin a century ago (Martin 1914, pp. 504–518), or if it needs to be subordinated to the more lasting components of organismal quantifications that have strict analogues in the biophysical sciences. Procrustes distance would then be reserved for the more cognitively dominated parts of biology, those that involve perception on the part of animals or systematists. For now, here in the early twenty-first century, we need to leave this question open.

### Parting Thoughts

Phrased in its most general terms, the problem with which this essay has been grappling is the mismatch between the scientific styles of the twentieth century and the information-processing styles of the 21st. Today’s standard GMM techniques inadvertently pursue a devolution of quantitative biology to correlation and regression indistinguishable in spirit from what Fisher and Pearson were trying to do a century ago. In this mimesis, however, we appear to be sacrificing too much of the actual information content of organisms as it is being revealed in steadily more and more detail by the more advanced instruments of the twenty-first century. The problem is the statisticians’ as much as the biologists’. Statistics students are not taught the first half of the steps in a quantitative scientific investigation, which concern the careful design of instruments and the nature of the measurements they generate. For an earlier meditation on this topic see Bookstein (2014), Section 8.3.

Yes, correlation is not causation, and no, while Big Data have arrived in many fields, Big Insights have not (Harford December 2014). Nevertheless, looking backward half a century from a future decade, say in 2035, GMM may have come to be viewed as a very early attempt to generate a Big Data workflow for quantitative biology in the smallest (meaning, most regulated) possible compass. Yet it seems to have the same problems as every other domain of Big Data. GMM sometimes would seem to be answering questions, but is quite incompetent at asking them; and while its data can submit to pattern engines, GMM is terrible at drawing intelligent distinctions among the resulting claims as regards their generalizability or their consilience over alternate modes of measurement.

Today’s most serious challenge to GMM is thus the requirement that it sharpen its rhetoric of answers, and likewise its rhetoric of questions, by incorporating as many as possible of the broken symmetries reviewed here: that is to say, the prior information afforded by the embedding of GMM within the toolkit of the quantitative biosciences *sensu lato.* As you have seen, there are techniques already in place for this purpose, some that are modifications of previously “standard” GMM and others that modify techniques useful in other quantitative fields. But we don’t know yet how to use them for the generation of reliable knowledge about organismal form. The challenge that GMM poses to multivariate analysis, in short, is the problem of knowledge transfer between whole scientific domains. Is the framework of matrices, linear combinations, and Euclidean rotations adequate for this task, or do we need a far more advanced ontology for this purpose? I will be eager to revisit this essay 10 or 15 years from now, in order to survey the innovations that, I trust, will have responded to its criticisms and requisites.

## Figures and Tables

**Fig. 11:**
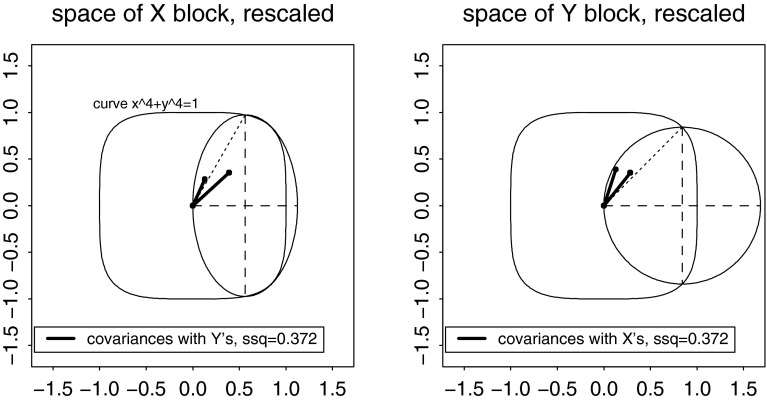
Geometry of the *RV* coefficient for two blocks of two measures each. The *rounded square* in each frame is the *curve* $x^4+y^4=1$ that normalizes the trace of the *square* of either $2\times 2$ covariance matrix. *Each ellipse* is aligned with its axes *horizontal* and *vertical* and the end of the shorter axis running through the center (0, 0) of the *quartic curve*. Then each is scaled so that its “diagonal”—the vector from the end of one axis to the end of the other—lies upon the quartic. With this joint scaling, the *RV* coefficient is the sum of the squared lengths of the heavy vectors out of (0, 0) in either figure: the vectors representing either the rows (*left*) or the columns (*right*) of the normalized covariance matrix $z_Xz_Ys_{PC_{Xi}PC_{Yj}}$. The text argues that this computation is without biological meaning

**Fig. 12:**
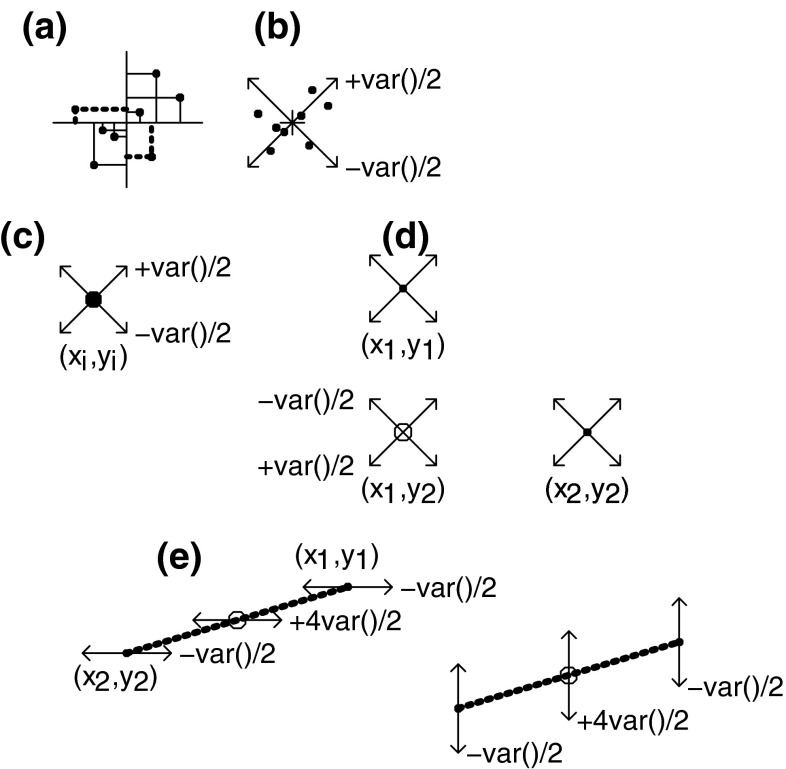
The diverse geometries of shape coordinate covariances. **a** The usual definition: cov(*x*, *y*) is the average of the signed areas of the rectangles drawn. (Areas of rectangles drawn in *dashed line* are taken with a minus sign.) Every *rectangle* includes the centroid of all the points (*x*, *y*) as its fourth corner. **b** Equivalent characterization as half the difference of two variances, that along the (1, 1) diagonal of the scatter grid and that along the $(-1,1)$ diagonal. Rotating the coordinate system of this diagram by $90^\circ$ reverses the sign of the covariance. **c** For the two shape coordinates of a single landmark point the scheme in panel (**b**) is adequate. **d** The covariance of any *x*-coordinate with any *y*-coordinate reduces to the geometry of a new, fictitious composite landmark, *open circle*, that combines the chosen *x*-coordinate with the chosen *y*. **e** The covariance of two *x*-shape coordinates (*left*) is an entirely different matter that begins with the other covariance identity, the excess of the variance of an average of two quantities (*open circle*) over half the sum of their variances separately. Rotating the coordinate system of *this* construction by $90^\circ$ does not change the sign of anything, but results in an entirely different construction (*right*), the covariance of the corresponding pair of *y*-shape coordinates

## Appendix 1: Example of A Symmetry Critique: The RV Formula

As an example of the type of critique that section “Rotations, Especially Their Basis in Covariance Structures” recommends be routinely applied to morphometric computational protocols, consider the formula for the *RV* statistic of Escoufier (Robert and Escoufier 1976), which is often touted (foolishly, in my judgment) as a tool for use in studies of “integration” or “modularity.” If *X* and *Y* are two blocks of measurements, say, *X*, $n\times p,$ and *Y*, $n\times q,$ on the same *n* cases, then the quantity under consideration is

$$\begin{aligned} RV(X,Y)\equiv \Sigma _i\Sigma _j s_{X_iY_j}^2\Big / \sqrt{\bigl (\Sigma _i\Sigma _j s_{X_iX_j}^2\bigr ) \bigl (\Sigma _i\Sigma _j s_{Y_iY_j}^2\bigr )} \end{aligned}$$

where $S_{XX}=(s_{{X_i}{X_j}})$ is the $p\times p$ covariance matrix of the *X*’s, $S_{YY}$ is the same for the *Y*’s, and $S_{XY}=(s_{{X_i}{Y_j}})$ is the $p\times q$ covariance matrix of the *X*’s by the *Y*’s. The letters *RV* stand for “R vectorielle,” meaning that this analogue of the familiar correlation coefficient seemed to Escoufier (1973) to be more reasonable than standard correlation-based methods for vector contexts (of which GMM is one).

Some useful identities from the multivariate textbooks (e.g., Mardia et al. 1979) apply here. For any matrices *A*, $n\times p,$ and *B*, $p\times n,$ we have

$$\begin{aligned} \mathrm{tr}~(AB)=\mathrm{tr}~(BA)=\Sigma _i\Sigma _j A_{ij}B_{ji}, \end{aligned}$$

where “tr” is the *trace operator,* sum of the diagonal elements of a square matrix.

Then, using the notation $'$ to refer to the transpose of a matrix,

$$\begin{aligned} \mathrm{tr}~(AA')=\mathrm{tr}~(A'A)=\Sigma _i\Sigma _j A_{ij}^2,\\ \mathrm{tr}~(RAR')=\mathrm{tr}~(AR'R)=\mathrm{tr}~(A) \end{aligned}$$

for *A* square and *R* a rotation, and thus

$$\begin{aligned} \Sigma _i\Sigma _j s_{{X_i}{X_j}}^2 = \mathrm{tr}~(S_{XX}^2) = \mathrm{tr}~(RDR'RDR')= \mathrm{tr}~(DD)= \Sigma _i d_i^2 \end{aligned}$$

for $$S_{XX}=RDR',$$$$D=R'S_{XX}R$$ diagonal, $$d_i=\sigma _{PC_i}^2$$. Also,

$$\begin{aligned} \mathrm{tr}~((R_1AR_2)(R_1AR_2)')=\mathrm{tr}~(R_1AAR_1')=\mathrm{tr}~(AA'), \end{aligned}$$

for any two rotation matrices $R_1,~R_2,$ and thus if we write *S* in the form of its *singular-value decomposition*$S=UDV',$*U* orthonormal $p\times p,$*V* orthonormal $q \times q,$ and *D* diagonal with nonzero entries $d_i$, a vector of length min(*p*, *q*),  then we have

$$\begin{aligned} \Sigma _i\Sigma _j s_{{X_i}{Y_j}}^2 = \mathrm{tr}~(S_{XY}S_{YX}) = \mathrm{tr}~(UDV'VDU')=\mathrm{tr}~(UDDU')= \mathrm{tr}~(DD)= \Sigma _i d_i^2 \end{aligned}$$

in this setting as well. Here each entry $d_i$ of the diagonal matrix *D* is the covariance of $XU_{.i}$ and $YV_{.i},$ the linear combinations of the *X*-variables and the *Y*-variables with coefficients given by the *i*th columns of *U* and *V*, respectively. So the numerator $\Sigma \Sigma s_{X_iY_j}^2$ of *RV*, considered on its own, is the sum of the squares of the latent variable covariances $d_i$ from the usual two-block Partial Least Squares analysis. Of course the usual PLS procedure involves the inspection of the singular vectors individually, not of their singular values squared and summed.

There is no geometrical constraint relating the *X*’s and the *Y*’s, so we can diagonalize each set separately. With this pair of basis choices, the traces $\mathrm{tr}(S_{XX}^2)$ and $\mathrm{tr}(S_{YY}^2)$ that are multiplied to give the square of the denominator of *RV* become the sums of squares of the corresponding principal component variances (summed squared eigenvalues—sums of fourth powers of their standard deviations), while the numerator of *RV* becomes the sum of squares of all *pq* crossproducts of each principal component score of the *X*’s by each principal component score of the *Y*’s.

Several features of this formula are already apparent.

1. That the analysis is rotatable is a bug, not a feature. No matter what information we might have about constraints on rotations of the measurements within the *X*-block or the *Y*-block—the direction of gravity, for example, or the presence of a growth-gradient from anteromedial to posterolateral—it cannot be accommodated as an influence on the computation.
2. The analysis does not produce *any* pattern descriptors of the original scores. We cannot inspect a rank-one approximation of $X'Y$ to see if its left and right singular vectors (columns of *U* and *V* in the PLS formulation) correspond to any sensible weighting schemes for the variables composing the blocks separately.
3. Sums of squared covariances are intuitively inaccessible, and likewise sums of fourth powers of standard deviations (of the principal components). The sum of the squared variances and covariances of the *X*’s or the *Y*’s has, in general, no familiar statistical setting; likewise the sum of the squares of all their crosscovariances. We know how to compare independently observed variances, by *F*-test, and we know how to deal with principal component variances, by recourse to the corresponding distribution of full covariance matrices (the *Wishart distribution,* see, e.g., Jolliffe 2002, Chapter 3); but what on earth are we supposed to do with sums of *squares* of their variances?
4. These quantities are likewise biologically inaccessible. What kind of numerical property of a sample of specimens is $\Sigma \Sigma s_{X_iY_j}^2$? Are its changes a meaningful descriptor of the action of any factor of form? selective gradient? genetic basis? Analogously, what is the meaning of $\Sigma _i(\sigma _{PC_i})^4$? How would we compare two of these values, as, for instance, for a wild type and a laboratory knockout strain? Comparisons of principal components require attention to the directions of those components, not just their variances; how can we make any sense of a quantity that completely ignores this directionality?

Some tentative answers to these questions can be pursued in the $2\times 2$ setting—two blocks of two variables each—by treating the data analysis question as a morphometric one in its own vector space. Indeed the geometry of the analysis, Fig. 11, can be diagrammed as a peculiar variant of a classic morphometric layout, the analysis of a single shape change tensor. Draw two ellipses with axes horizontal and vertical, their axes standing for the principal components of the *X*-block and the *Y*-block with semi-axis lengths set to the standard deviations of those components. For each one, draw the line (dotted in the figure) that is the vector sum of the two semi-axes—that is, the diagonal of the rectangle in which that ellipse would be inscribed. Then, in a maneuver that is bizarre by any conventional multivariate logic, scale each hyperellipse by the careful inscription of its semi-axis diagonal in the curve $x^4+y^4=1$ (a curve that is sometimes called a *hyperellipse*) in the manner shown in the figure. Write the scaling factors as $z_X,z_Y.$

The matrix $S_{XY}$ is, after the rotation and scaling to these axes, a $2\times 2$ matrix of covariances $z_Xz_Ys_{PC_{Xi}PC_{Yj}}$. It can be imagined as composed of two rows, each of which can be drawn as a vector out of (0, 0) in the plane of the *Y*-ellipse, or, equivalently, two columns, each of which can be drawn as a vector on the *X*-ellipse. *With this scaling* (which I have already argued makes no biological sense) the *RV* coefficient is just the sum of the squared lengths of either set of vectors.

This expression is geometrically unfamiliar, and puzzling. If those little vectors were orthogonal, then *RV* would equal the squared length of the hypotenuse of the right triangle they generated; but usually they are not orthogonal, so that interpretation is blocked. And if the ellipses were circles, then each vector would stand for the multiple regression of its principal component on the axes of the other system, and its length would be proportional to the $R^2$ of the corresponding multiple regression, up to a scaling; but the ellipses are not circles (in general—in the figure, the right-hand one happens to be one), so *that* interpretation is blocked. The *RV* coefficient apparently ignores the angle between those vectors—this seems an unreasonable loss of information, even though either of the vectors can be reflected in either of the axes without change of meaning (since principal components “point both ways”)—and also ignores any variation in length of the vectors whose squared lengths are being summed. Crosscovariance matrices $z_Xz_Ys_{PC_{Xi}PC_{Yj}}$ that evaluate to $$\left( \begin{array}{cc}.7071&{}0\\ 0&{}0\end{array}\right) $$, $$\left( \begin{array}{cc}.5&{}0\\ 0&{}.5\end{array}\right) $$, and $$\left( \begin{array}{cc}.3&{}.4\\ .3&{}.4\end{array}\right) $$ between a pair of trace-normalized $2\times 2$ blocks all generate the same value 0.5 for the *RV*, an equivalence that seems quite misleading inasmuch as the corresponding morphometric reports would be wholly divergent in every other aspect of a description. (For instance, the first and third of these can be reduced to one single pair of crosscorrelated dimensions, but the second example requires two such pairs to be explained.)

The visualization in the general case, *p* dimensions against *q*, is a straightforward conceptual generalization of this same diagram. We rotate each block to its own principal axes and “draw” its covariance structure via a hyperellipsoid scaled so that its semi-axis diagonal falls upon the surface $\Sigma x_j^4=1$ (a hyperquadric). With this scaling, the *RV* coefficient equals the sum of squared lengths of all of the vectors expressing either the rows (on the left hyperellipsoid) or the columns (on the right hyperellipsoid) of the original crosscovariance matrix of the PC’s of the *X*-block by those of the *Y*-block. The angles among these vectors are simply ignored, as is any variation among their lengths. In an application where the *X*-block, for example, consists of shape coordinate data, there is no distinction available regarding which components of the vectors on the right correspond to *x*-coordinates and which to *y*-coordinates, or regarding how the lengths and orientations of the vectors on the left assort with respect to the positions and adjacencies of the corresponding landmarks upon the form. Interpretation of the analysis will be even more difficult when *both* blocks consist of shape coordinates. Closely spaced landmarks are usually correlated for biological reasons, but we cannot tell the extent to which the pattern of crosscovariances conforms to this aspect of their covariances structures separately or instead cuts across them. And surely it matters if a pattern of crosscovariances, when expressed in the bases of the blocks’ own principal components separately, is more like $$\left( \begin{array}{cccc}d_1&{}0&{}0&{}0\\ 0&{}d_2&{}0&{}0\\ 0&{}0&{}d_3&{}0\\ 0&{}0&{}0&{}d_4\end{array}\right) $$ or like $$\left( \begin{array}{cccc} 0&{}0&{}0&{}d_1\\ 0&{}0&{}d_2&{}0\\ 0&{}d_3&{}0&{}0\\ d_4&{}0&{}0&{}0\end{array}\right) $$—whether the vectors in our analysis tend to be longer in the direction of the hyperellipses’ long axes or not. But the *RV* coefficient is oblivious to such considerations.

In other words, the *RV* ratio makes no sense in terms of any reasonable biological interpretations of these axes, ellipses, or covariances. The actual *value* of the *RV* coefficient is not itself the answer to any reasonable biological question. (You have probably guessed that already from the weird form of that superquadric in the figure, which is quite different from the ellipses we are accustomed to exploit in linear multivariate analysis.) The *RV*’s single role (in the eyes of those who consider it to merit any role at all) seems merely to be significance testing. This, however, as I have commented at length elsewhere [see Bookstein (2014), Section 4.3.4] is not any sort of a finding but only a formulaic answer to the question of “whether we may publish the claim of an association” between the *X*- and *Y*-blocks of variables, i.e. whether, in the usual bureaucratic idiom, “further investigation is indicated.” But that decision, like every other instance of significance-testing in biology, is merely an aspect of the sociology of the academy, having nothing to do with proper modes of scientific inference at all.^10^

Thus even though the scheme of two blocks of variables, each one rotated separately to a basis of its own principal components, is sometimes intuitively accessible, the formula for the *RV* coefficient itself is not. From a detailed examination of the geometry corresponding to the formula it can be seen that the computed *RV* does not answer any natural query about the explanations that might be associated with the two lists of variables it is describing. It should be obvious by now that the *RV* formula should not be used in connection with landmark data, and particularly not if the data include any semilandmarks, because the arbitrary spacing of those points will render all of the preceding concerns even more intractable. The *RV* coefficient thus supplies a nearly perfect example of the fallacy of inappropriate symmetries with which this article is concerned.

### Appendix 2: Diagrams for the Covariances of Shape Coordinates

It is not only the caustic critiques, like the preceding Appendix about the RV coefficient, that should explicate formulas by way of their geometry. It would be good to have such translations for supportive didactic texts as well. This Appendix provides a graphical table of geometrical equivalents for the common currency of the text’s geometric morphometric models, the covariance structure of a set of shape coordinates. The development can be followed in the panels of Fig. 12.

You probably first encountered the idea of a covariance via its role as the numerator of the standard formula for the regression coefficient. If the line $y=ax+b$ is to serve as the least-squares fit to a scatter of data points $(x_i,y_i),$ then the slope *a* must be equal to the ratio of the covariance of *x* and *y* to the variance of *x*: the formula

$$\begin{aligned} a = {{\sum _{i=1}^n (x_i-\overline{x})(y_i-\overline{y})}/n\over {\sum _{i=1}^n (x_i-\overline{x})^2/n}} \equiv {{\mathrm{cov}(x,y)}\over {\mathrm{var}(x)}}. \end{aligned}$$

It was Fisher who named this numerator the *covariance* of *x* and *y* by analogy with the already-standardized name, *variance,* for its denominator, the selfsame formula when *x* and *y* are set to the same vector of measured values.

For the purpose of the graphics to follow it is helpful to write down two elementary identities dealing with this product-moment:

$$\begin{aligned} \mathrm{cov} (x,y) = \mathrm{var}((x+y)/2)-\mathrm{var}((x-y)/2), \end{aligned}$$

and

$$\begin{aligned} \mathrm{cov}(x,y)={1\over 2}\bigl (\mathrm{var}(x+y)-\mathrm{var}(x)-\mathrm{var}(y)\bigr ). \end{aligned}$$

Panel (a) in Fig. 12 illustrates our initial definition of the covariance—the average value of the crossproduct $(x-\overline{x})(y-\overline{y})$ is, after all, identically equal to the average (signed) area of the rectangles shown. As noted in the main text, in the general case this already raises certain issues of biological meaning. Does it make any sense to imagine a unit of “area” for the general product of two measurements, for instance, blood pressure by net litter weight? But in the applications to geometric morphometrics with which this paper is concerned, the variables whose covariances are under inspection are shape coordinates that are technically dimensionless (cm/cm), and so the issue of the composite unit describing products like these is seemingly circumvented.

Under a slightly more general assumption—units of *x* and *y* identical, if not necessarily dimensionless—the definition of the covariance can be rearranged into the form shown in panel (b), half the difference of variances of the main diagonals of the scatter grid. We know that covariance reverses when the sign of either variable reverses: cov$(x,-y)$ = −cov(*x*, *y*). But also, since cov(*x*, *y*) is the same as cov(*y*, *x*),  we can achieve the same effect by just rotating the coordinate system of the figure here by $90^\circ .$ (The role of those two diagonals reverses, so they swap signs in the formula.) It follows from elementary calculus that there must be at least one intermediate orientation of the coordinate system (the alignment with their own pair of principal axes) for which this covariance between the two shape coordinates of a single pair is exactly zero. Rotations notwithstanding, the construction here explicates one of the three main types of shape coordinate covariance, the covariance between the two shape coordinates of a single landmark, as notated in panel (c).

But there are two other types of shape coordinate covariance, those between the *x*-shape coordinate of one landmark and the *y*-shape coordinate of another, panel (d), and those between two *x*-shape coordinates or two *y*-shape coordinates, panel (e). The construction for the mixed (*x*, *y*) case is not so different from that for the two shape coordinates of one single landmark. In the figure, the landmarks at which the two coordinates in question originated are marked with a small solid dot, and the (*x*, *y*) combination of interest is taken as a proper pair of coordinates of one single new point, the one marked at the big open disk, which simply pairs the *x*-shape coordinate of the first landmark with the *y*-shape coordinate of the second. This is order-dependent: the value of cov$(x_1,y_2)$, shown, is not necessarily the same as the value of cov$(x_2,y_1)$, the other pairing.

The situation is entirely different for the third case, the covariance of a pair of parallel shape coordinates (two *x*’s or two *y*’s). Panel (e) diagrams this by exploiting the other covariance identity, the one about midpoints. The covariance we seek is half of the weighted sum of three different parallel directional variances: four times the variance of an average (the open disk), minus the sum of the variances of the contributing coordinates separately (the two small solid dots).

From the contrast between panel (d) and panel (e) we learn something important about the multivariate statistics of shape coordinates in the large. In panel (d), the covariance of interest is the difference of two directional variances. The kind of shape transformation that would make this value particularly large and positive, and hence a major contributor to some summary pattern description of the covariance structure of the shape coordinate scheme as a whole, would be one that increases the variance along the diagonal with the positive coefficient: increasing $x_1,$ say, while simultaneously increasing $y_2.$ Such a factor evidently involves effects in two directions at $90^\circ$ at the two landmarks. But for landmarks positioned as in panel (d), the combination of an increment in $x_1$ with an increment in $y_2$ actually serves to *shorten* the separation vector between them. It seems an odd sort of morphogenetic factor that would move two distant landmarks in perpendicular directions, increasing their distances from other landmarks while at the same time shortening the specific distance between them. In comparison, consider the sort of factor that would result in an increase of the covariance cov$(x_1,x_2),$ panel (e). To move these landmarks in parallel is not necessarily to shorten any other distances. So this is a likelier sort of factor to turn up in a morphogenetic toolkit. Any process that moves the two landmarks in parallel will increase the variances of each of the three points in the diagram to the same extent, and so will increase this particular weighted sum by the multiple $(4-1-1)/2=1$ of that same additional variance.

Of these two ways of generating covariance between shape coordinates, the origin in parallel coordinates (*x* with *x* or *y* with *y*) seems thereby more natural, biologically speaking, than the origin in disparate coordinates, an *x* with a *y*. Running the inference the other way, parallel shape coordinates are much likelier to have been strongly and jointly affected by large-scale form factors than nonparallel ones: *the form factors of largest scale will typically be found to operate along the long diameters of the underlying form,* and to relatively stretch or shrink them rather than giving them a relative rotation.

(By using the notation of advanced calculus this can be phrased in a somewhat more physical language, the *Helmholtz decomposition* of the vector field mapping the points of one organism’s image to the points of another’s. Covariances like cov$(x_1,x_2)$ are tuned to the transformations characterized by nonzero **divergence**$\nabla \cdot \Phi ,$ where $\Phi$ is some scalar summary of a pattern of expansion and $\nabla$ is the differential operator (*d*/*dx*, *d*/*dy*, *d*/*dz*); but the covariances like cov$(x_1,y_2)$ are tuned to transformations of nonzero **curl**$\nabla \times \mathbf{A,}$ where **A** is a different sort of mathematical creature, a “vector potential” the role of which is to generate terms in rotation, i.e., spin. Maxwell’s equations (of electrodynamics) notwithstanding, such transformations are much less commonly encountered in the course of morphogenetic explanations. Morphogenetically, strain is easier to imagine generating than torque; morphogenetic processes are much easier to imagine that displace landmark locations away from one another than that rotate landmark-to-landmark segments with respect to one another.)

The specific maneuver that transforms the original Cartesian coordinates into Procrustes shape coordinates heightens this contrast between the two families of covariances. If the $x_1$ and $x_2$ in panel (e) happen to lie at opposite ends of a diameter of the form, then after a Procrustes superposition the variance of their average is likely to be nearly zero, so the covariance of the two coordinates will approximately equal its maximal *negative* value, leading to a principal component in the form of a contrast between the two original coordinates, i.e., an estimated (relative) length of the diameter they span. It is exactly this abstract overweighting of the largest-scale aspects of shape variation that the deflation technique of section “Deflated Procrustes Analysis” was designed to intercept. At the same time, the rotation step of the Procrustes superposition acts to *attenuate* torques around the centroid like those generating large values of covariances in panel (d), and thereby to downweight one or the other of the directional variances var$(x_i\pm y_j)$ in that panel that would otherwise be of commensurate magnitude with terms of the form cov$(x_i,x_j)$ or cov$(y_i,y_j)$, the terms for the strains of largest scale.

The other main component of the current GMM toolkit, the thin-plate spline, concurs with this emphasis, in that the basic interpolant is a sum of terms that all have curl zero. Forcing a spline map to rotate a small region with respect to its surround requires a complicated finite-element process that does not approach a proper infinitesimal form: see Bookstein and Green (1993). (This is the main reason that the thin-plate spline is unsuited to the comparison of articulated linkages, for which relative rotation of parts is an essential component of actual biomechanical function.)

The organism is, so to speak, unaware of these covariances. The analyses in this Appendix and the panels of its figure are concerned not with actual organismal biology but rather with the way that covariances of shape coordinate structures might represent or misrepresent patterns pertinent *to* that organism. Taken together, they serve as a premonition that something is not quite right with the way we typically identify morphogenetic factors with statistical factors. It is exactly this unpleasant paradox—the unwanted empirical domination of features at large scale in our conventional GMM analyses—that the method of deflated Procrustes analysis, section “Deflated Procrustes Analysis”, was developed to circumvent.

## Footnotes

1 The problem arises often enough in a single dimension that there are two distinct mathematical special cases for handling it. In one setting, subscripts lie on the number line in natural order. For example, the matrix of expected distances between points connected by random walks of 1, 2, $\ldots$ steps of the same step variance can be assembled in a matrix whose entries form a *Toeplitz matrix*—they are constant along every line parallel to the diagonal, proportional to the absolute value of that difference of subscripts. For an application in evolutionary biology, see Bookstein (2013b). The other mathematical case is analogous except that the subscripts lie on a circle: the so-called *circulant* matrix model. The psychometrician Louis Guttman was particularly interested in methods for analyzing matrices derived from such lists of variables. In still other models, *both* the rows and columns are circular: this is the so-called *torus model* for dihedral angles of DNA chain backbones studied in its statistical aspects by K. V. Mardia and colleagues.

2 The connection between optimal variances and zero covariances technically applies only to “flat” descriptor spaces. For the general Riemannian manifold, the different characterizations of PC’s are no longer equivalent, and their empirical use in such fields as neuroanatomy is consequently a great deal more fraught.

3 Since Centroid Size is $x_1^2+y_1^2+x_2^2+\ldots ~,$ one has $d\left( x_1^2+y_1^2+x_2^2+\ldots \right) \propto x_1dx_1+x_2dx_2+\ldots ~$; this is the projection of the observed variation of shape coordinates $(dx_1,dy_1,dx_2,\ldots )$ onto the vector $(x_1,y_1,x_2,\ldots )$ that is the fourth row of *J*.

4 This was a property of the landmark points themselves before it pertained to these numbers. As I said in Bookstein (1991, page 61), “Landmarks are the points at which one’s explanations of biological processes are grounded. $\ldots$ Landmark-based morphometrics is the embodiment within biometrics of the functional form of biological explanation.”

5 For any sample of homologous landmark configurations in two dimensions, the *nonuniform component* of variation can be approximated as the result of projecting out a version of the *J*-matrix, section “Procrustes Distance, Procrustes Coordinates”, that has two further rows $(cy_1,dx_1,cy_2,dx_2,\ldots ,cy_k,dx_k)$ and $(-dx_1,cy_1,-dx_2,cy_2,\ldots ,-dx_k,cy_k),$ where $c=\Sigma x_i^2/\sqrt{\Sigma x_i^2 (1-\Sigma x_i^2)}$ and $d=(1-\Sigma x_i^2)/\sqrt{\Sigma x_i^2 (1-\Sigma x_i^2)}$. Because of the constraint $\Sigma x_i^2+\Sigma y_i^2=1$ imposed in the course of standardizing *J*, this extended version is still orthonormal (all rows of length 1, all crossproducts 0). The projection *on* these two additional rows is an estimate of the *uniform component* of any such shape change around the Procrustes average. See Bookstein (2014):418, equation (7.1) and subsequent text. In 3D the analogous projection involves five dimensions, not just two.

6 Hence for a Mantel regression along these lines to have any inferential weight in a study using GMM there must be a consideration of broken symmetries like those along with all the other aspects of symmetry-breaking reviewed in section “Four Ubiquitous Problems”. In particular, however comfortable it may feel to announce that “forms sampled at close spacing are more similar than those sampled at wide spacing,” a Mantel test based on a morphological distance, whether Procrustes distance, Mahalanobis distance, or any other variant, is almost certainly unjustified in its arithmetic.

7 Consider, for instance, scaling the effect of some constant load on an anatomically variable shell with respect to the shell’s thickness *h*. From the basic Rayleigh equation, treating that thickness *h* as a parameter, we have ${\text{elastic}}\; {\text{energy}}\sim Ah({\text{extension}})^2 + Bh^3({\text{bending}})^2$ for appropriate constants *A* and *B*, so the two additive components scale differently with thickness—the transformation from Procrustes coordinates to elastic deformation cannot be affine (linear). Also, while the squared “extension” in this equation is approximately proportional to squared Procrustes uniform distance plus the squared change in log Centroid Size, the “bending” being squared is the bending geometry within the shell only, not extended over all space as in the thin-plate formalism. The variety of available quantifications of image deformation is particularly well-reviewed in Grenander and Miller (2007).

8 The analysis there included cetacean forms, which I have omitted from the present analysis because the blowhole has so completely rearranged the skull. Also, the data set had one missing value, for the hyena, and so for convenience I have omitted that specimen also. There remain 55 taxa under consideration. For the present didactic purpose it is sufficient to attend only to the thirteen midline landmarks (there were also eleven bilateral pairs).

9 But the extensions must be restricted to the *quantitative* domains among our neighboring disciplines. There are much weaker forms of prior knowledge—“modularity,” embryonic layers, etc.—that correspond only to *qualitative intuitions* about homologies in diverse evo-devo processes. Hypotheses of “common origin” are rarely specific enough to generate the sort of quantitative regularity claim that would plausibly be allowed to break a prior geometric symmetry.
